# Utilizing Monocarboxylate Transporter 1‑Mediated Blood–Brain Barrier Penetration for Glioblastoma Positron Emission Tomography Imaging with 6‑[^18^F]Fluoronicotinic Acid

**DOI:** 10.1021/acs.molpharmaceut.5c00457

**Published:** 2025-07-03

**Authors:** Pyry Dillemuth, Abiodun Ayo, Tomi T. Airenne, Petter Lövdahl, Emel Bakay, Xiaoqing Zhuang, Heidi Liljenbäck, Sami Tuomas Paunonen, Jonne Kunnas, Pauliina Filppu, Johan Rajander, Mark S. Johnson, Anne Roivainen, Tiina A. Salminen, Jessica M. Rosenholm, Pirjo Laakkonen, Xiang-Guo Li

**Affiliations:** † Turku PET Centre, 8058 University of Turku, Turku FI-20520, Finland; ‡ Department of Chemistry, University of Turku, Turku FI-20500, Finland; § Translational Cancer Medicine Research Program, Faculty of Medicine, 3835 University of Helsinki, Helsinki FI-00290, Finland; ∥ iCAN Flagship Program, University of Helsinki, Helsinki FI-00290, Finland; ⊥ Structural Bioinformatics Laboratory and InFLAMES Research Flagship Center, Biochemistry, Faculty of Science and Engineering, 1040 Åbo Akademi University, Turku FI-20520, Finland; # Pharmaceutical Sciences Laboratory, Faculty of Science and Engineering, Åbo Akademi University, Turku FI-20520, Finland; ¶ Accelerator Laboratory, Åbo Akademi University, Turku FI-20520, Finland; ∇ Turku PET Centre, Turku University Hospital, Turku FI-20520, Finland; ○ InFLAMES Research Flagship, University of Turku, Turku FI-20520, Finland; ⧫ Turku Center for Disease Modeling, University of Turku, Turku FI-20520, Finland; †† Laboratory Animal Centre, HiLIFE University of Helsinki, Helsinki FI-00290, Finland

**Keywords:** fluorine-18, glioblastoma, G protein-coupled receptor GPR109A, monocarboxylate transporter 1, niacin, nicotinic acid

## Abstract

Glioblastoma is the most malignant brain tumor in adults, and its prognosis remains dismal. The blood–brain barrier impedes the effectiveness of many drugs, which are otherwise effective for cancer treatment. Monocarboxylate transporter 1 (MCT1) is expressed on endothelial and glioblastoma cells. Our approach aims to leverage MCT1 to transport theranostic agents across the blood–brain barrier. In this context, we present herein the application of fluorine-18-labeled nicotinic acid (denoted as [^18^F]­FNA) for glioblastoma imaging using positron emission tomography (PET). An intracranial mouse model of human glioblastoma was prepared by using patient-derived BT12 cells. PET imaging, ex vivo biodistribution, brain tissue autoradiography, and tumor and tissue uptake kinetic analyses were performed. Additionally, the ligand–target interaction was studied using in silico modeling. The xenografted glioblastomas were distinctly visualized in all 18 mice with a mean standardized uptake value of 0.92 ± 0.11 and tumor-to-brain ratio of 1.66 ± 0.17. The tumor uptake of intravenously administered [^18^F]­FNA decreased by 76% on average when MCT1 was inhibited, whereas preadministration of 60 mg/kg niacin significantly enhanced [^18^F]­FNA tumor uptake. The G protein-coupled receptor GPR109A is a high-affinity receptor for niacin (nicotinic acid). In silico simulations indicated that both niacin and fluorinated nicotinic acid (FNA) interact with the GPR109A receptor in a similar manner. In the presence of a GPR109A inhibitor in in vivo experiments, the tumor residence of [^18^F]­FNA was extended. [^18^F]­FNA has demonstrated its potential for PET imaging in a clinically relevant orthotopic glioblastoma model, and MCT1 plays a crucial role in [^18^F]­FNA transport. The results pave the way for the development of niacin-derived theranostics for glioblastoma care.

## Graphical abstract

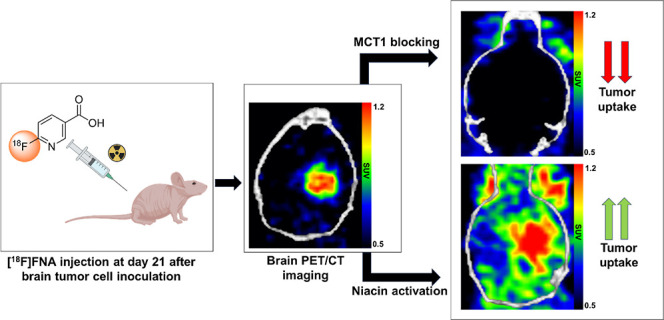

## 1 Introduction

Glioblastoma is the most common and aggressive primary malignant brain tumor in adults. Even with the current standard care, the median survival of patients remains dismal, only about one year.^1^ No breakthrough in glioblastoma treatment has been introduced since 2005 when the Stupp treatment regimen was launched.^2^ The blood–brain barrier (BBB) impedes the efficacy of many drugs, including antibody-based checkpoint inhibitors.^3^ To deliver agents through the BBB for imaging-based diagnostics or treatment purposes, one strategy is to use the transporters available in the BBB. Monocarboxylate transporter 1 (MCT1) is expressed in the brain endothelial cell membrane and is upregulated in glioblastoma tumor cells.^4–6^ Particularly interesting, MCT1 is overexpressed in glioblastoma tumor stem-like cells,^7^ a subpopulation of tumor cells responsible for tumor recurrence and treatment resistance. The MCT1 expression influences drug absorption and bioavailability^8^ and is associated with cancer metastasis.^9^ MCT1 is a highly relevant target for cancer treatment, and several drugs, including the MCT1 inhibitor AZD3965 (Figure 1), are currently under clinical evaluation.

**Figure 1:**
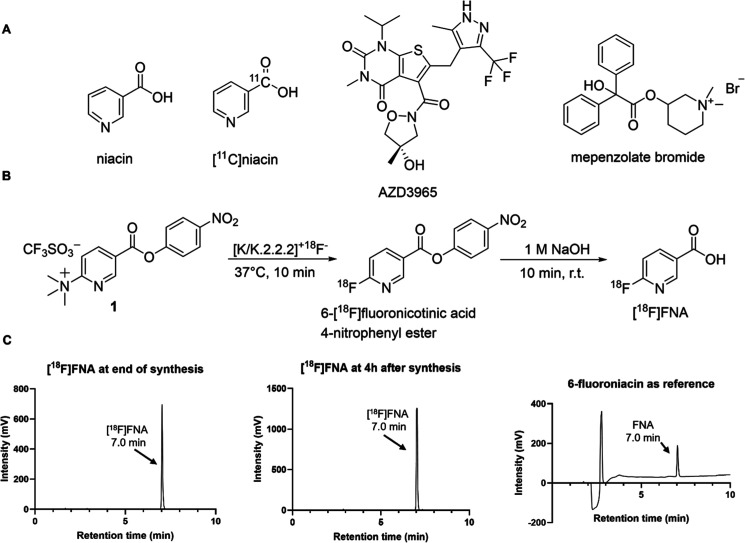
Chemical structures and schemes. (A) Chemical structures of niacin, [^11^C]­niacin, AZD3965, and mepenzolate bromide. (B) Radiosynthesis chemical scheme of [^18^F]­FNA. (C) HPLC chromatographs of the end product [^18^F]­FNA at the end of synthesis, 4 h after the synthesis under radioactivity detection, and the nonradioactive reference 6-fluoroniacin under UV detection.

We have set out to develop radiopharmaceuticals for the following purposes: for noninvasive whole-body molecular imaging of MCT1 expression dynamics in disease and health, for patient stratification for MCT1-targeted treatment, for monitoring treatment-response in MCT1-targeted drug development with positron emission tomography (PET), and for MCT1-mediated radiotheranostics. In this part of the work, we aim to develop a fluorine-18 radiolabeled niacin analogue (6-[^18^F]­fluoronicotinic acid, abbreviated as [^18^F]­FNA) for PET imaging of glioblastoma and as a tool to visualize MCT1-mediated BBB penetration (Figure 1). Bongarzone et al. prepared carbon-11-labeled niacin ([^11^C]­niacin) and performed PET imaging studies in healthy C57BL/6 mice.^10^ In general, ^11^C-labeled molecules maintain biological properties identical to those of their corresponding unlabeled counterparts. However, the physical half-life of ^11^C is only 20.3 min, which poses constraints in its clinical applications and off-site use. Fluorine-18 is one of the most useful radionuclides for PET applications. It has a physical half-life of 109.7 min, which makes it suitable for prolonged clinical and preclinical procedures for research purposes and enables long-distance logistics to research sites. Herein, we report the radiosynthesis and characterization of [^18^F]­FNA and its PET studies in mice with intracranial human glioblastoma and evaluate MCT1-mediated transport through the BBB. Additionally, we have attempted to clarify the interaction between [^18^F]­FNA and G protein-coupled receptor GPR109A, one of the high-affinity receptors of natural niacin.^11^ In humans, GPR109B is a low-affinity receptor of niacin, in addition to GPR109A.

## 2 Experimental Section

### 2.1 Materials and General Methods

The precursor molecule *N*,*N*,*N*-trimethyl-5-((4-nitrophenoxy)­carbonyl)-pyridin-2-aminium trifluoromethanesulfonate (**1**) for the preparation of the intermediate compound 6-[^18^F]­fluoronicotinic acid 4-nitrophenyl ester (Figure 1) was custom-synthesized by R & S Chemicals (Kannapolis, NC, USA). The primary antibodies anti-GPR109A polyclonal rabbit (Invitrogen, PA5-90579), anti-GPR109B polyclonal rabbit (Invitrogen, PA5-106832), and anti-MCT1 polyclonal rabbit (Invitrogen, bs-10249R) were purchased from Thermo Fisher Scientific (Waltham, MA, USA). The pig anti-rabbit HRP-conjugated secondary antibody (P0217) used for Western blot analysis was purchased from DAKO (Jena, Germany). The donkey anti-rabbit AlexaFluor 488-conjugated secondary antibody (Invitrogen, A21206) used for immunofluorescence staining was purchased from Thermo Fisher Scientific. The methods for in silico homology modeling and docking analyses are described in the Supporting Information.

### 2.2 Radiosynthesis of [^18^F]­FNA

The chemical scheme for the radiosynthesis of [^18^F]­FNA is shown in Figure 1, and the radiosynthesis was performed with a custom-made remote-controlled device. The radiolabeled intermediate compound 6-[^18^F]­fluoronicotinic acid 4-nitrophenyl ester was prepared similar to previous literature studies.^12,13^ In-house produced [^18^F]­fluoride (3.8–5.2 GBq) was extracted onto a Sep-Pak Accell Plus QMA Plus Light anion-exchange cartridge (Waters, Milford, MA, USA), which was preconditioned with 0.5 M aqueous potassium carbonate (2.0 mL) and water (5.0 mL) (TraceSELECT, Honeywell, Charlotte, NC, USA). Elution of [^18^F]­fluoride was carried out using a solution of Kryptofix 2.2.2 (9.2 mg, 23.1 μmol) and potassium carbonate (1.6 mg, 15.4 μmol) in Milli-Q water (76.9 μL) and acetonitrile (1.9 mL). The eluate was then dried under nitrogen flow at 120 °C, followed by the addition of the precursor compound **1** (10.0 mg, 22.2 μmol) in 0.8 mL of acetonitrile. The reaction mixture was maintained at 37 °C for 10 min and then diluted with water (1.0 mL). The intermediate compound 6-[^18^F]­fluoronicotinic acid 4-nitrophenyl ester was purified using high-performance liquid chromatography (HPLC) equipped with a radioactivity detector and a reversed-phase C18 column (Jupiter Proteo, 250 × 10 mm, 5 μm, 90 Å; Phenomenex, Torrance, CA, USA) at a flow rate of 4 mL/min. Solvent A was 0.1% trifluoroacetic acid (TFA) in water, while solvent B was 0.1% TFA in acetonitrile. The HPLC elution gradient was from 45 to 70% B during 0–13 min. The HPLC fraction containing 6-[^18^F]­fluoronicotinic acid 4-nitrophenyl ester was collected and diluted with 30 mL of water, and the product was extracted onto an Oasis HLB Plus Light cartridge (Waters, Milford, MA, USA). 6-[^18^F]­Fluoronicotinic acid 4-nitrophenyl ester was then hydrolyzed on-cartridge by slowly passing 1.0 M NaOH (580.0 μL) through the HLB cartridge. The NaOH solution was kept in the cartridge for 10 min, after which [^18^F]­FNA was eluted from the cartridge with 2.0 mL of water into a vial containing 2.0 M HCl (140.0 μL) and 2.0 M phosphoric acid (125.0 μL). The chemical identity and radiochemical purity of [^18^F]­FNA were examined using HPLC. Accordingly, 0.5–0.8 MBq of [^18^F]­FNA was injected into a C18 reversed-phase column (Jupiter Proteo, 250 × 4.6 mm, 5 μm, 90 Å; Phenomenex). The results were then compared to cold reference HPLC results (Figure 1), where 20–50 nmol of commercial 6-fluoronicotinic acid (Merck, Rahway, NJ, USA) in water was used. Solvent A consisted of 0.1% TFA in water, while solvent B contained 0.1% TFA in acetonitrile. The HPLC elution gradient began at 10% B and ended at 50% B between 0 and 10 min, at a flow rate of 1.5 mL/min, and was monitored by radioactivity detection and UV detection at wavelengths of 220 and 254 nm. The shelf life of [^18^F]­FNA was measured by taking samples from the final product at time points up to 4 h and analyzed with HPLC similarly as described above for radiochemical purity measurements. The distribution coefficient Log *D*_7.4_ was measured by first adding 5 kBq of [^18^F]­FNA to a mixture of 600 μL of phosphate-buffered saline (PBS, pH 7.4) and 600 μL of 1-octanol. The mixture was then thoroughly mixed and the layers were separated by centrifuging for 3 min at 12,000*g*. After separation, aliquots of 400 μL were taken from each layer and the radioactivity was measured using a well counter (Wizard 2. 3″ model 2480 automatic gamma counter, PerkinElmer Wallac Oy, Turku, Finland). The test was performed in triplicate. The Log *D*_7.4_ value for [^18^F]­FNA was calculated with radioactivity decay correction using the following formula:

$$\mathrm{Log}\;D=\log_{10}\frac{counts\;in\;the\;octanol\;phase}{counts\;in\;the\;PBS\;phase}$$

To measure the molar activity of the end product [^18^F]­FNA, 0.05, 0.20, 0.50, 1.00, and 2.00 nmol of nonradioactive reference compound FNA samples were examined using HPLC with UV detection. A calibration curve was established to link the amount of FNA with the UV absorbance areas. Each sample was measured in triplicate. The end product [^18^F]­FNA samples were also analyzed with HPLC, and the UV absorbance served as the input function to determine the molar amount of the end product. Molar activity was determined by dividing the total radioactivity by the total molar amount of the end product in the whole batch. The results were reported as GBq/μmol at the end of the synthesis. The HPLC methods used were as follows: samples were injected into a C18 reversed-phase column (Jupiter Proteo, 250 × 4.6 mm, 5 μm, 90 Å; Phenomenex). Solvent A was 0.1% TFA in water, while solvent B was 0.1% TFA in acetonitrile. The HPLC elution gradient was from 10% to 50% B during 0–10 min at a flow rate of 1.5 mL/min, and UV detection was performed at a wavelength of 254 nm.

### 2.3 Glioblastoma Cell Cultivation and Mouse Model Preparation

The BT11, BT12, and BT27 cell lines originated from glioblastoma patients’ surgical samples were obtained as described previously.^14^ Briefly, BT12, BT11, and BT27 glioblastoma stem cell lines were cultivated in serum-free and 2 mM l-glutamine and 2.4 g/L sodium bicarbonate-rich DMEM/F12 (Dulbecco’s modified Eagle medium/nutrient mixture F-12) containing medium (Gibco, New York, NY, USA) supplemented with 2% B-27 (Gibco, Skjern, Denmark), 15 mM 4-(2-hydroxyethyl)-1-piperazineethanesulfonic acid (HEPES) buffer (Fisher Scientific, Leicestershire, UK), 100 units/mL penicillin and 0.1 mg/mL streptomycin, 0.01 μg/mL recombinant human fibroblast growth factor FGF-b (Peprotech, London, UK), and 0.02 μg/mL recombinant human epidermal growth factor EGF (Peprotech). S24 and ZH305 glioblastoma cells were cultured in a phenol red-free neurobasal medium (Gibco) containing 2% B-27 supplement without vitamin A (Thermo Fisher), 100 units/mL penicillin and 0.1 mg/mL streptomycin, 0.01 μg/mL recombinant human fibroblast growth factor FGF-b, and 0.02 μg/mL recombinant human epidermal growth factor EGF. All cell lines were maintained in 5% CO_2_ in a humidified incubator at 37 °C.

Immunocompromised female mice (Rj: NMRI-FOXn1nu/nu strain, 6–7 weeks old, Janvier Laboratories, Le Genest-Saint-Isle, France), were intracranially engrafted with BT12 cells.^14^ The glioblastoma cells (0.5 × 10^5^) in 5 μL of 0.9% saline were inoculated into the right hemisphere of the mouse brain with a Hamilton syringe using a stereotaxic device (World Precision Instruments, Sarasota, FL, USA). The inoculation site was 2 mm distance from the bregma and 2.5 mm deep into the brain parenchyma. Mice were placed under 2–3% isoflurane anesthesia and kept warm at 37 °C using a heating plate and a rectal probe connected to a temperature control system (World Precision Instrument) throughout the procedure. For analgesia, mice were administered with 0.01 mg/kg buprenorphine and 5 mg/kg carprofen before and after intracranial inoculation, respectively, and continued for 2 days postoperation. The mice were used for PET studies at day 21 post tumor cell inoculation. All animal work was approved by the National Project Authorization Board in Finland with the permission number of ESAVI/10262/2022 and was carried out in compliance with the EU Directive 2010/EU/63 on the protection of animals used for scientific purposes.

### 2.4 PET Imaging, Ex Vivo Biodistribution, and Autoradiography

The animal study design is shown in Figure S1. In addition to the mice with glioblastoma, immunocompromised female mice (Rj: NMRI-FOXn1nu/nu strain, 6–7 weeks old, Janvier Laboratories, Le Genest-Saint-Isle, France) without tumors were used as control mice. The PET study of the control mice was performed only with the radiotracer [^18^F]­FNA, without the administration of any blocking or activation agents. All of the mice had ad libitum access to food and water prior to the PET study. PET imaging was performed in combination with high-resolution computed tomography (HRCT) for an anatomical reference and attenuation correction on Molecubes small-animal PET and CT imaging systems (Molecubes NV, Gent, Belgium) for mice. During the whole imaging procedure, animals were under anesthesia with a continuous inhalation of 1–2% isoflurane and heating. The animals were first imaged with HRCT for 6 min and then injected with [^18^F]­FNA (4.73 ± 0.22 MBq for 42 mice) intravenously (i.v.) via tail vein cannulation. Two mice were imaged at a time, and 60 min dynamic PET data were collected in a list mode. In the in vivo blocking, inhibition, or activation experiments, the imaging procedures were similar except that the mice were i.v. administrated with AZD3965 (1.1 mg/kg of bodyweight), mepenzolate bromide (5 and 15 mg/kg of bodyweight), or niacin (15 and 60 mg/kg of bodyweight) via a tail vein 15 min before the administration of [^18^F]­FNA. PET data were reconstructed with an ordered subset expectation maximization 3-dimensional algorithm (OSEM-3D) into 6 × 10 s, 4 × 60 s, 5 × 300 s, and 3 × 600 s time frames, and CT was reconstructed using iterative image space reconstruction algorithm (ISRA). PET/CT images were analyzed with the Carimas 2.10 software (Turku PET Centre, Finland, www.turkupetcentre.fi/carimas/). Regions of interest (ROI) were defined with the spline tool referring either to the anatomical CT image (whole tissues) or to the focal maximum (tumor) in the brain PET image. Brain ROIs were ROIs at approximately the contralateral sites of the tumor ROIs with similar volumes. Quantitative results were expressed as standardized uptake values (SUVs), tumor-to-brain ratio (i.e., target-to-background ratio, TBR), and time–activity curves (TACs). A correct ROI placement was confirmed by trans-axial and sagittal views. SUV was normalized for the injected radioactivity dose and the animal body weight.

Immediately after PET imaging, mice were euthanized under deep anesthesia by a cardiac puncture via the left ventricle, followed by cervical dislocation. Subsequently, the mice were perfused with 10 mL of PBS via the left ventricle to remove radioactivity in the blood. Tissue samples were collected and counted with a Wizard gamma counter (Wallac Oy, Turku, Finland). The measured radioactivity was normalized with the injected radioactivity dose per animal weight, the weights of the tissue samples, and radioactivity decay. The injected dose was corrected for residual radioactivity in the tail and the cannula. The results were expressed as a percentage of the injected radioactivity dose per gram (% ID/g) of the tissue or percentage of the injected radioactivity dose (% ID). Mouse brains were snap-frozen in isopentane cooled with dry ice and prepared as cryosections with thicknesses of 20 and 10 μm. The cryosections with 20 μm thickness were thaw-mounted onto microscopy glass slides and exposed to a phosphor imaging plate (BAS-TR2025, Fujifilm, Tokyo, Japan) overnight inside lead shielding. Digital autoradiographs were acquired with a BAS-5000 scanner (Fuji, Tokyo, Japan) and analyzed with Carimas software. The results were expressed as photostimulated luminescence per square millimeter (PSL/mm^2^) with a background correction. After autoradiography, the same tissue sections were stained with standard hematoxylin and eosin (H&E) staining protocols in the Histology Core Facility at the University of Turku, Finland.

### 2.5 Blood Radioactivity Analysis and In Vivo Stability of [^18^F]­FNA

At the end of PET imaging (60 min postinjection of [^18^F]­FNA), blood samples were collected from mice into heparinized tubes. Blood cells and plasma were isolated by centrifugation (2100*g*) for 5 min. Plasma proteins were precipitated by adding an equal volume of acetonitrile and separated from the plasma by centrifugation (14,000*g* for 3 min) at r.t. The radioactivity of the isolated blood components was measured with a Wizard gamma-counter. Protein-free plasma supernatant samples were analyzed by HPLC equipped with a radioactivity detector. The analysis was done on a reversed-phase C18 column (Phenomenex, Jupiter Proteo, 250 × 10 mm, 5 μm, 90 Å) at a flow rate of 5 mL/min. Solvent A was 0.1% TFA in water and solvent B was 0.1% TFA in acetonitrile. The HPLC elution gradient during 12 min was from 15% B to 50% B. Additionally, mouse brain samples were collected into Eppendorf tubes and homogenized together with acetonitrile using tissue grinders (Fisher Scientific, Hampton, NH, USA). The solid brain matter was separated by centrifugation (14,000*g* for 3 min) at r.t. and the resulting clear acetonitrile supernatant was analyzed with HPLC using the same method as described above for protein-free plasma samples.

### 2.6 Immunofluorescence Staining

Ten-micrometer thick frozen tissue sections were fixed in 4% paraformaldehyde (Histolab) for 10 min and washed in 1× PBS (pH 7.4) for 15 min. Tissue sections were permeabilized in 1× PBS containing 0.3% Triton X-100 for 5 min and blocked in 1× PBS containing 0.03% Triton X-100 and 1% bovine serum albumin (BSA) for 60 min. Tissue sections were incubated for 24 h at 4 °C with an anti-MCT1 rabbit polyclonal antibody (bs-10249R, Bioss Antibodies, MA, USA) and Cy3-conjugated human specific monoclonal anti-vimentin (C9080, Sigma), prepared in blocking buffer at 1:400 and 1:800 dilutions, respectively, followed by a 10 min wash with 1× PBS. The sections were incubated with a goat-anti-rabbit Alexa Fluor 647-labeled secondary antibody (A-21244, Thermo Fisher Scientific, USA) for 2 h at 37 °C, and counterstained with 2-(4-amidinophenyl)-1*H*-indole-6-carboxamidine (DAPI) for cell nuclei visualization. Samples were mounted on coverslips using a Mowiol solution (Sigma-Aldrich). Images were acquired with a digital slide scanner (Pannoramic P1000, 3DHistech Ltd., Budapest, Hungary).

### 2.7 Western Blot Analysis

Cells were lysed in 1% NP-40 lysis buffer [(50 mM Tris–HCl, 150 mM NaCl, 1% NP-40 (Cal Biochemicals, Hyderabad, India), pH 7.4)] containing freshly added protease inhibitors (EDTA-free complete, Roche, Basel, Switzerland) and phosphatase inhibitors (PhoSTOP phosphatase, Roche) for 30 min on ice. The cell debris was removed at 14,000*g* maximum speed for 20 min at 4 °C using centrifuge 5425R (Eppendorf SE, Hamburg, Germany). Protein quantitation was performed by using a Pierce BCA protein assay kit (Thermo Fisher). The lysates were diluted in 2× Laemmli sample buffer (62.5 mM Tris–HCl, 2% sodium dodecyl sulfate (SDS), 25% glycerol, pH 6.8) containing 15% β-mercaptoethanol (Life Technologies Corporation, Paisley, UK) and bromophenol blue (Sigma-Aldrich, Saint Louis, MO, USA). The samples were heated at 95 °C for 5 min and 10 μg of the protein was loaded to a 4–20% Tris–glycine gel (Invitrogen, Waltham, MA, USA) followed by transferring onto a polyvinylidene fluoride (PVDF) membrane in a 1× transfer buffer (Bio-Rad) using a Transblot Turbo instrument (Bio-Rad) by following manufacturer’s instructions. The membrane was incubated in blocking buffer (1× tris-buffered saline (TBS) containing 5% BSA and 0.1% Tween 20) for 60 min at 37 °C, probed with the primary antibody after an overnight incubation at 4 °C. The next day, the membrane was subjected to a 20 min wash with 1 × TBS + 0.1% Tween 20, and 10 min wash with 1× TBS followed by probing with the horseradish peroxidase (HRP)-conjugated secondary antibody for 2 h at 37 °C. The protein bands were visualized with a Pierce ECL Western Blotting substrate (Thermo Scientific) and Azure 500 imaging system (Azure Biosystems, Dublin, CA, USA).

### 2.8 Statistical Analysis

Statistical analyses were performed using GraphPad Prism version 10. Results were expressed as the mean ± standard deviation. The unpaired Student’s *t*-test was used to measure differences between independent data sets. *P* values <0.05 were considered statistically significant.

## 3 Results

### 3.1 Radiopharmaceutical Chemistry

[^18^F]­FNA was prepared in decay-corrected radiochemical yields of 38.1% ± 9.7 (*n* = 9) and the radiochemical purity was 99.7% ± 0.6 at the end of the synthesis (Figure 1C). The stability of [^18^F]­FNA in the final formulation was at least 4 h at room temperature (23 °C), and the chemical identity was confirmed by HPLC analysis of the reference 6-fluoronicotinic acid. The molar activity was 146.7 ± 63.6 GBq/μmol at the end of the synthesis. The total synthesis time was 112 ± 9.2 min, starting from the end of the bombardment. The pH of the end product was between 5.0 and 7.4. The distribution coefficient Log *D*_7.4_ was −2.23 ± 0.01 (*n* = 3).

### 3.2 PET/CT Imaging and Brain Tissue Autoradiography

Intracranial glioblastoma xenografts were clearly visualized in all 18 mice (Figure 2). The skull bone and other tissues in the head showed low radioactivity concentrations. The mean standardized uptake values (SUV_mean_) for the tumor and healthy brain were 0.92 ± 0.11 (*n* = 18) and 0.55 ± 0.06 (*P* < 0.0001), respectively. The maximum SUV (SUV_max_) values for the tumor and healthy brain were 1.54 ± 0.25 (*n* = 18) and 1.26 ± 0.12 (*P* < 0.0001). The tumor-to-brain ratio (tumor SUV_mean_/brain SUV_mean_, abbreviated as TBR) was 1.66 ± 0.17 (*n* = 18) at 5–10 min postinjection, at which time point the tumor uptake reached the highest level. The healthy mice of the same strain were used as controls, and no focal radioactivity uptake was observed in the corresponding brain areas where tumor cells would have been inoculated (Figure 2). The PET imaging experiments were performed using different batches of mice, different batches of [^18^F]­FNA, and different researchers on different days. Hence, the PET imaging results were reproducible. Representative whole-body PET/CT images are shown in Figure 3. To assess the radioactivity uptake in tumor-containing brain tissue samples, after PET imaging, mouse brains were collected and cryosectioned into slices. Cryosections were subjected to autoradiography, and a high focal radioactivity uptake was observed (Figure 3B), which corresponded to the tumor areas as judged by the hematoxylin–eosin (H&E) staining of the same tissues (Figure 3C). The radioactivity concentration in the tumor area at 60 min after a tracer injection was 60.8 ± 22.8 photostimulated luminescence per square millimeter (PSL/mm^2^, *n* = 8) and TBR was 2.0 ± 0.2.

**Figure 2:**
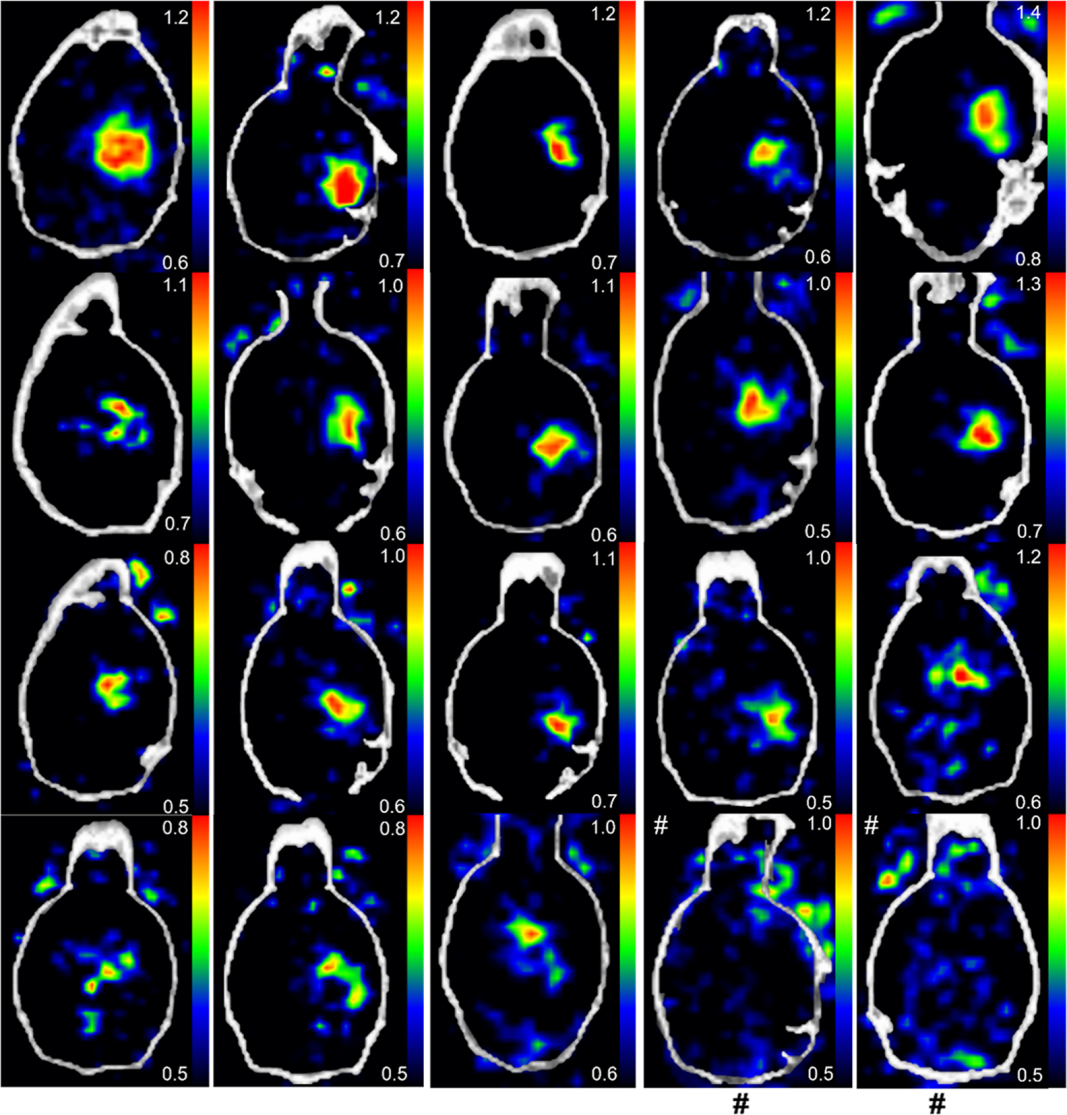
PET/CT images of mouse brains with or without glioblastomas. The PET images are the time-weighted means of frames from 4 to 30 min postinjection of [^18^F]­FNA. Glioblastoma was clearly visualized in all 18 mice. The last two PET/CT images (marked with #) are examples from two healthy control mice. The color scale bar in each image indicates the SUV_mean_.

**Figure 3:**
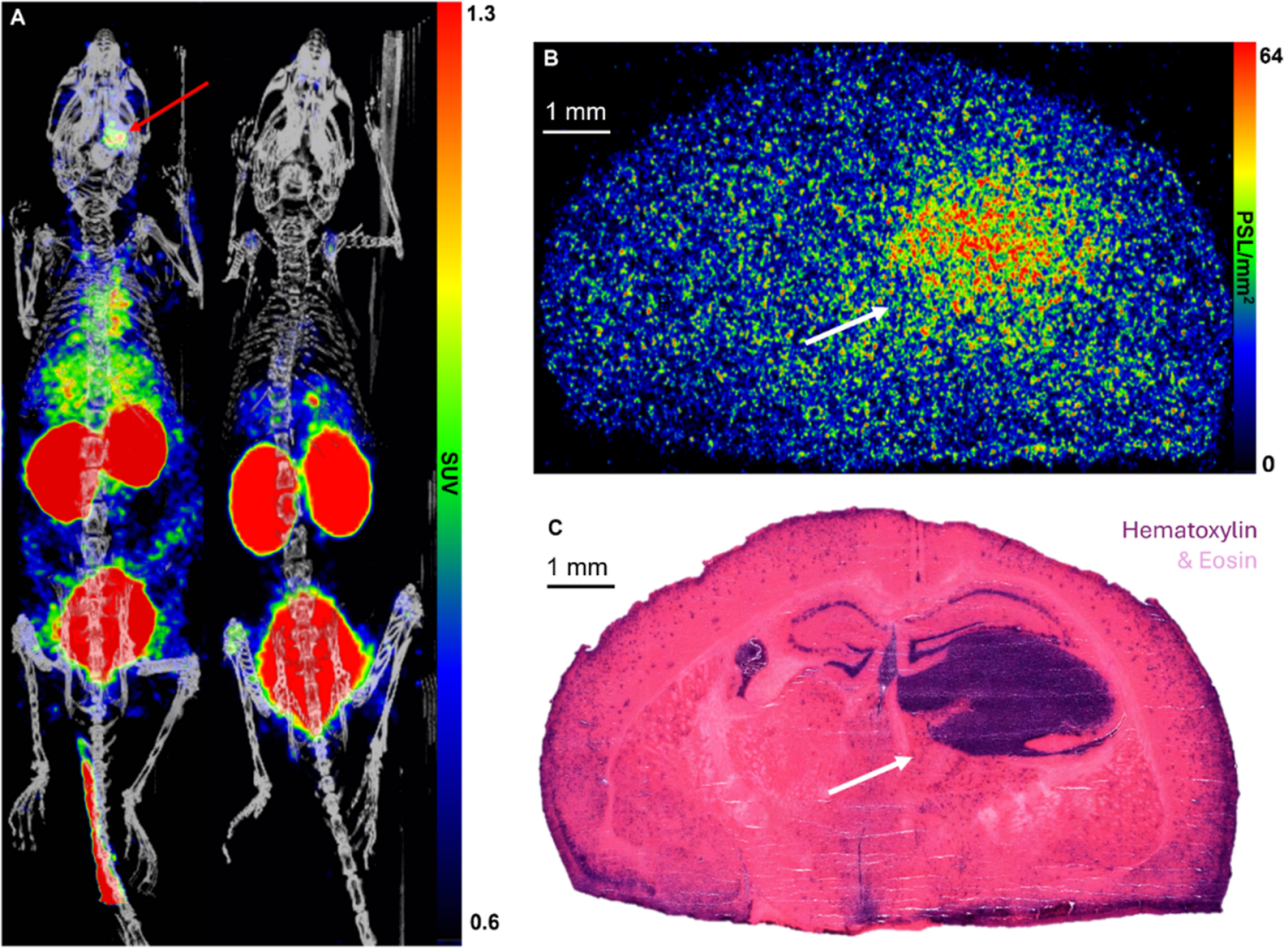
(A) Representative whole-body [^18^F]­FNA PET/CT images of glioblastoma-bearing mice without MCT1 blocking (on the left, tumor indicated by red arrows) and with MCT1 blocking (on the right). PET images are the time-weighted means of frames from the 4–30 min postinjection of [^18^F]­FNA. (B) Autoradiography and (C) H&E staining of glioblastoma-containing brain tissue sections (white arrows indicate the tumor).

### 3.3 Ex Vivo Biodistribution of [^18^F]­FNA

The biodistribution study included 18 tissue samples, plasma, urine, and blood cells (Figure S2, Table S1), and the data was expressed as % ID/g of tissue weight. In addition, the % ID values for some of the organs are presented in Table S2. The whole brain uptakes in mice with glioblastoma were 0.43 ± 0.09% ID/g (*n* = 4) and 0.19 ± 0.05% ID. The highest radioactivity concentration (% ID/g) was observed in the urine (431.43 ± 129.28) and kidneys (5.69 ± 1.03), indicating that [^18^F]­FNA was mainly excreted via the urinary tract. The lowest uptake was found in the pancreas (0.21 ± 0.11), lungs (0.22 ± 0.09), and brown fat (0.24 ± 0.08). The skull bone and femur (bone + marrow) uptake was also relatively low (1.13 ± 0.12 and 1.30 ± 0.56, respectively), indicating no significant in vivo ^18^F-defluorination. In general, the uptake was low (≤1.12) in all other tissues and blood. In healthy control mice, the major excretion route was via the urinary tract, as expected. The whole brain uptakes were 1.05 ± 0.13% ID/g (*n* = 4) and 0.48 ± 0.07% ID.

### 3.4 Inhibition of MCT1 Blocks [^18^F]­FNA Uptake

As shown in Figure 4A, tumors were not visualized by PET in the presence of the MCT1 blocker, AZD3965. The [^18^F]­FNA uptake in tumors was decreased by 76%, and the radioactivity uptake in the whole brain was significantly lower than in the nonblocking experiment. In the presence of AZD3965, the brain SUV_mean_ was 0.20 ± 0.02 (*n* = 4), which was significantly lower than that in the nonblocking experiments where SUV_mean_ of brain was 0.60 ± 0.06 (*n* = 18, *P* < 0.001). In the non-blocking experiments, the radioactivity uptake in the tumor reached the highest level during 5–10 min postinjection and then slowly declined during the next 50–55 min of PET (Figure 4D). In the MCT1 blocking experiments, the shape of the time–activity curves (TACs) differed from that of the nonblocking experiments and nearly plateaued after the first 2 min. The blocking effect was also confirmed by ex vivo autoradiography and gamma counting of tissues. MCT1 inhibition at an AZD3965 dose of 1.1 mg/kg clearly decreased tumor uptake from 60.8 ± 22.8 PSL/mm^2^ (nonblocking group) to 15.2 ± 5.5 PSL/mm^2^ (MCT1 blocking group, *n* = 4) with a *P* < 0.001. These results suggest that MCT1 plays a major role in [^18^F]­FNA transport into glioblastoma and the transport is rapid.

**Figure 4:**
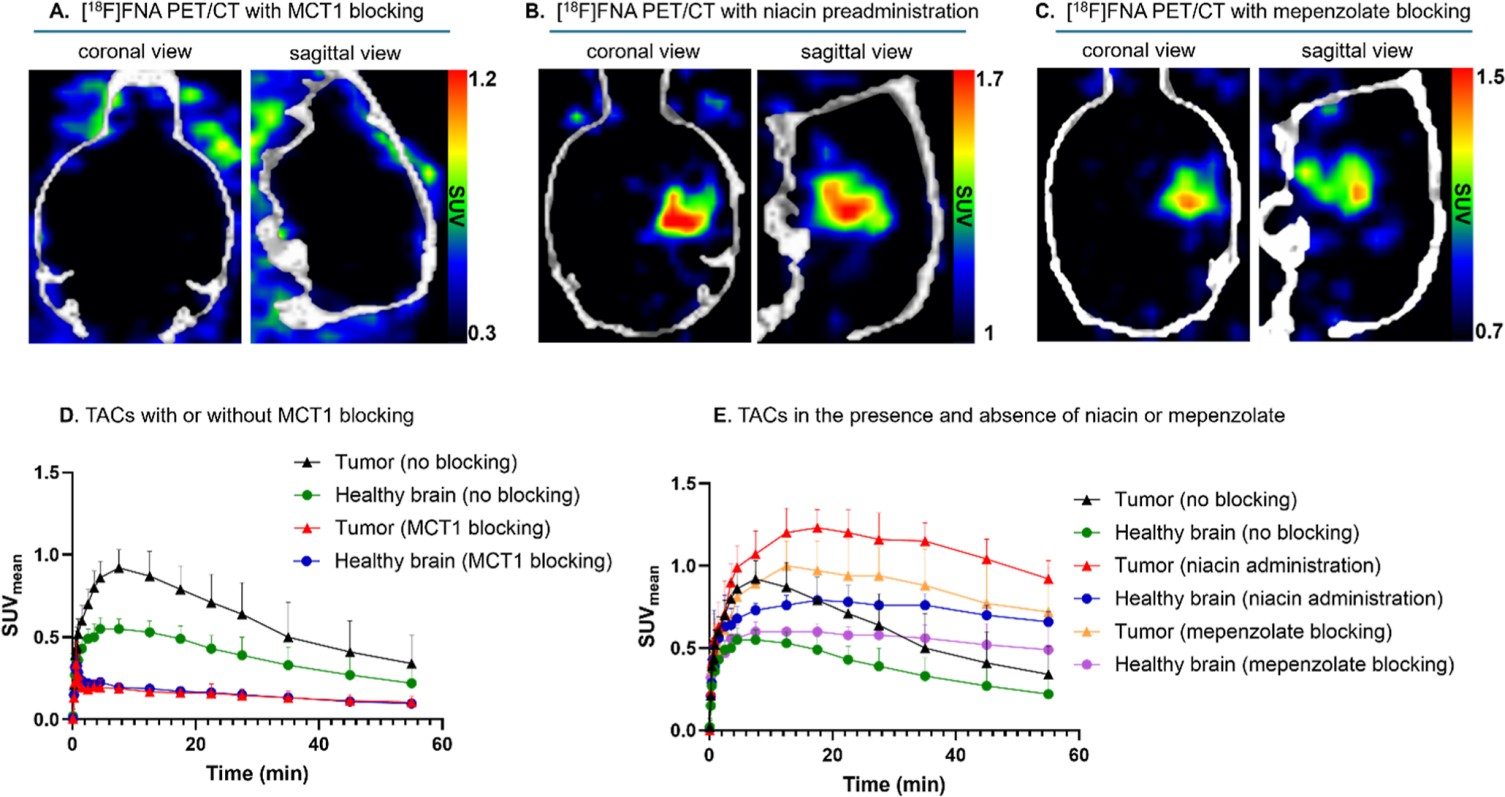
MCT1 blocking and GPR109A activation and inhibition experiments in mice bearing intracranial glioblastoma. (A) PET/CT images of glioblastoma-bearing mice in the presence of the MCT1 inhibitor AZD3965 (1.1 mg/kg). (B) PET/CT images in the presence of niacin (60 mg/kg bodyweight). (C) PET/CT images in the presence of GPR109 inhibitor mepenzolate bromide (15 mg/kg bodyweight). (D) Comparison of TACs with and without MCT1 blocking in mice with glioblastoma. (E) Comparison of TACs in the absence or presence of niacin (60 mg/kg) or in the presence of GPR109A inhibitor, mepenzolate bromide (15 mg/kg).

The MCT1 blocking effect was clearly observed not only in the glioblastoma and the brain but also in brown fat, harderian glands, heart, kidneys, lungs, small intestine, and stomach wall (Figure 5; Table S1). In all of those tissues, the differences in the radioactivity uptake were statistically significant, and the biggest difference was observed in brain tissue samples (*P* < 0.01). However, there was no statistically significant difference in the skull bone and femur uptake between the MCT1-blocked and nonblocking groups.

**Figure 5:**
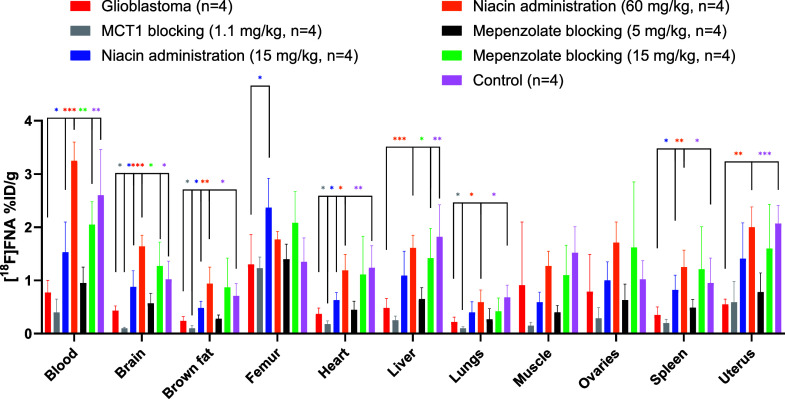
Ex vivo biodistribution of [^18^F]­FNA in mice with or without a glioblastoma xenograft. Statistical differences between the groups are indicated as *P < 0.05, **P < 0.01, and ***P < 0.001. No markings are made if there is no statistically significant difference. Asterisks indicating a statistical significance are color-coded according to the groups.

### 3.5 Niacin Preadministration Enhances [^18^F]­FNA Tumor Uptake and Retention

Niacin is an essential vitamin in the human body and in ingested foods. Next, we studied whether the niacin intake influences the [^18^F]­FNA tumor uptake. To study the effect of excess niacin on the [^18^F]­FNA uptake in glioblastoma and other tissues, mice (*n* = 4 each group) were i.v. administered with niacin at doses of 15 or 60 mg/kg 15 min before [^18^F]­FNA injection. The tumor SUV_mean_ was 0.87 ± 0.12 (*n* = 4) at a 15 mg/kg dose and 1.20 ± 0.14 (*n* = 4) at a 60 mg/kg dose. The TBR were 1.43 ± 0.18 and 1.57 ± 0.11, respectively. At a dose of 60 mg/kg niacin preadministration, the tumor uptake was significantly higher than without niacin preadministration (*P* < 0.001). Niacin preadministration increased the tumor uptake by an average of 30%. Representative PET/CT images observed at a dose of 60 mg/kg are shown in Figure 4B, which showed striking brain tumor delineation. Interestingly, the trends of TACs in glioblastoma tumors and the brain were dramatically changed in the presence of excess niacin (60 mg/kg) compared with TACs without niacin preadministration (Figure 4E). Preadministration of niacin-enhanced [^18^F]­FNA retention and uptake in the tumor and healthy brain areas. According to the ex vivo tissue gamma-counting data (Figure 5; Table S1), the radioactivity uptake in most tissue samples was higher (*P* values <0.01 or <0.0001) compared with the group without niacin preadministration. For instance, the % ID/g of the brain was 0.43 ± 0.09 and 1.64 ± 0.21 (*P* < 0.001) in the absence and presence of niacin (60 mg/kg), respectively. Additionally, a statistically significant difference was observed between the groups administered with niacin at doses of 15 and 60 mg/kg, in several tissues including the skull bone, brain, muscle and brown fat.

### 3.6 Mepenzolate Preadministration Prolongs [^18^F]­FNA Tumor Retention

Niacin is a small organic compound, and the fluorinated analogue [^18^F]­FNA is not anticipated to have identical biological properties as niacin itself. One of the most studied receptors of niacin is GPR109A, and mepenzolate bromide is a known inhibitor of GPR109A.^11,15^ Thus, we next determined whether mepenzolate bromide preadministration influences the [^18^F]­FNA uptake in our BT12 glioblastoma mouse model. Accordingly, the same mice (*n* = 4) with glioblastoma were first PET/CT imaged with [^18^F]­FNA on the first day, and then on the second day PET/CT imaged with [^18^F]­FNA 15 min after the administration of mepenzolate bromide (5 or 15 mg/kg). The glioblastoma was still clearly visualized in the presence of mepenzolate bromide (Figure 4C). The tumor uptake reached the highest level at 10–15 min postinjection, and the SUV_mean_ of the tumor were 0.83 ± 0.07 (*n* = 4) at a dose of 5 mg/kg and 1.00 ± 0.15 (*n* = 4, *P* < 0.05) at a dose of 15 mg/kg. The TBR were 1.69 ± 0.12 and 1.68 ± 0.26 (*P* = 0.810), respectively. According to the TACs, [^18^F]­FNA was better retained in both tumor and healthy brain areas compared with the [^18^F]­FNA uptake in the absence of mepenzolate bromide (Figure 4E). When the amount of mepenzolate bromide was increased from 5 mg/kg to 15 mg/kg, the brain uptake increased from 0.57 ± 0.19 to 1.27 ± 0.45% ID/g (*P* < 0.01, Figure 5; Table S1).

### 3.7 [^18^F]­FNA Is Stable In Vivo

[^18^F]­FNA demonstrated excellent in vivo stability at 60 min postinjection. The proportion of the intact tracer of the total radioactivity was 97.5% ± 1.4 (*n* = 4). The HPLC chromatograms of the blood samples were compared against the [^18^F]­FNA standard (Figure S3A,C). A small radiometabolite peak was observed in the chromatogram at a retention time of 5.6 min. However, this metabolite was detected in only trace amounts and was not analyzed further. In addition, the in vivo stability of [^18^F]­FNA was assessed in brain homogenates 60 min postinjection, and the tracer was completely stable (Figure S3B). The radioactivity of blood components was assessed in healthy mice and glioblastoma-bearing mice. The glioblastoma mice received the [^18^F]­FNA tracer alone or with the preadministration of niacin (60 mg/kg), mepenzolate blocker (15 mg/kg), or MCT1 blocker (1.1 mg/kg) 15 min before tracer injection. The blood cell binding in the healthy control group was 28.7% ± 1.6 (*n* = 4), and in the glioblastoma mice 23.7% ± 1.4 (*n* = 4), 25.4% ± 2.9 (*n* = 4), 26.6% ± 1.0 (*n* = 4), and 37.9% ± 2.4 (*n* = 4), respectively. The plasma protein binding in the healthy control group was 6.6% ± 1.1 (*n* = 4), and in the glioblastoma mice 3.2% ± 0.5 (*n* = 4), 8.5% ± 0.5 (*n* = 4), 8.6% ± 2.1 (*n* = 4), and 7.5% ± 0.5 (*n* = 4), respectively.

### 3.8 MCT1, GPR109A, and GPR109B Expressions in Glioblastoma Subtypes

We studied the MCT1 protein expression in BT12 glioblastoma xenograft samples (Figure 6A). Part of the tumors expressed high amounts of MCT1. In addition, expressions of MCT1, GPR109A, as well as GPR109B proteins were studied in the patient-derived glioblastoma stem cell lines BT11, BT12, BT27, S24, and ZH305 by Western Blot analysis (Figure 6B). BT11 belongs to a classical subtype and is characterized by the expression of astrocyte genes, BT12 represents the mesenchymal glioblastoma type, and BT27, S24, and ZH305 represent the proneural glioblastoma subtype. MCT1 and GPR109A expressions were observed in all the five cell lines analyzed (Figure 6B), while GPR109B was detected in BT11, BT27, S24, and ZH305 cells but not in BT12. We also analyzed the MCT1 mRNA expression in the classical, mesenchymal, and proneural glioblastoma subtypes with the Ivy-Gap data set by using the GlioVis data visualization tool. MCT1 mRNA was expressed in all the three subtypes at similar levels (Figure 6C). The MCT1 mRNA expression was the highest in the pseudopalisading cells compared to other histological entities, including cellular tumor, infiltrating tumor, leading edge, and microvascular proliferating cells using the Ivy-Gap data set (Figure 6D).

**Figure 6:**
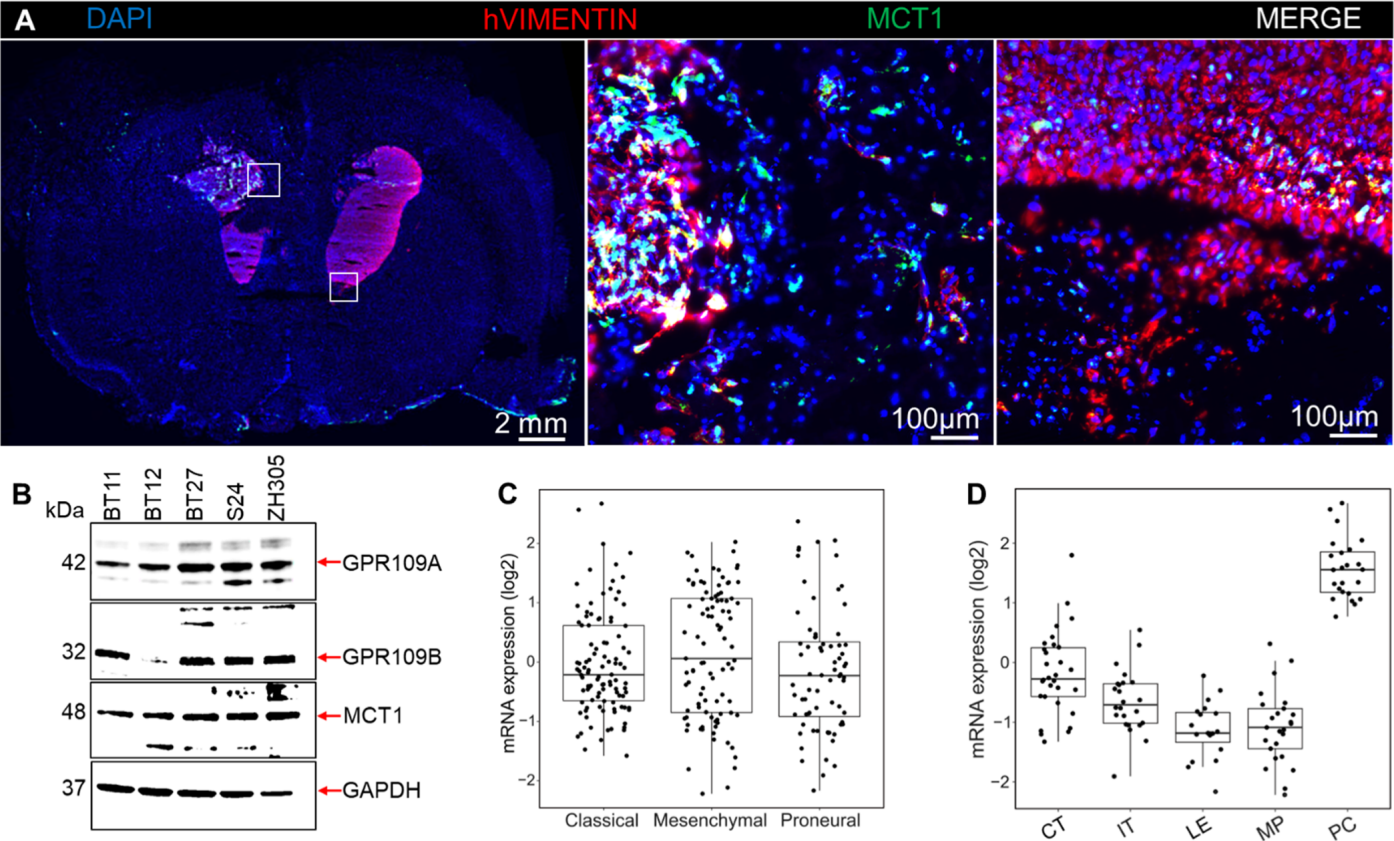
MCT1, GPR109A, and GPR109B expression in glioblastoma subtypes. (A) Immunofluorescence staining of the mouse brain tissue section with anti-MCT1 (green) and human specific anti-vimentin (red) antibodies. Cell nuclei were visualized with DAPI in blue color. The middle and right upper panels show higher magnifications of the white boxed areas. (B) Western blot analysis of GPR109A, GPR109B, and MCT1 protein expression in patient-derived glioblastoma cell lines BT11 (astrocytic), BT12 (mesenchymal), and BT27, S24, and ZH305 (proneural). Glyceraldehyde 3-phosphate dehydrogenase (GAPDH) was used as the loading control. (C) MCT1 mRNA expression in glioblastoma subtypes of classical (*n* = 103), mesenchymal (*n* = 93), and proneural (*n* = 72) in the Ivy_Gap data set. (D) Analysis of glioblastomas showing the MCT1 mRNA expression in the different anatomical structures of glioblastoma. Highest expression was detected in the pseudopalisading cells (*n* = 24). The graph also shows the expression in tumor cellular cells (cellular tumors, CT, *n* = 30), disseminated tumor cells (infiltrating tumor, IT, *n* = 24), leading edge (*n* = 19), and tumor-associated proliferating endothelial cells (microvascular proliferation, MP, *n* = 25) using the Ivy_Gap data set.

### 3.9 In Silico Interaction Simulation between GPR109A and 6-Fluoronicotinic Acid

As mepenzolate bromide preadministration prolongs the [^18^F]­FNA tumor residence, the question arose whether [^18^F]­FNA interacts with GPR109A similarly to natural niacin. Accordingly, we performed in silico simulation studies using the corresponding nonradioactive compound 6-fluoronicotinic acid and GRP109A structures (Figures 7; S4–S8). We examined the binding patterns of niacin and 6-fluoronicotinic acid to mouse (mGPR109A) and human (hGPR109A) receptors using homology modeling and molecular docking analyses. The results revealed that both ligands exhibited remarkably similar binding modes to hGPR109A and mGPR109A. This similarity likely extends to the rat receptor rGPR109A, which shares over 95% sequence with mGPR109A and maintains all amino acid residues involved in ligand binding. Despite the slightly different relative orientation of the fluorine atom of the tracer in the mGPR109A and hGPR109A structural models, i.e., variation in distances of the fluorine atom of 6-fluoronicotinic acid to the surrounding aromatic residues W91^ECL1^(hGPR109A)/W88^ECL1^(mGPR109A) and Y87^2.64^(hGPR109A)/Y84^2.64^(mGPR109A), the studied receptors are likely to strongly bind the [^18^F]­FNA tracer. A residue important in hGPR109A activation (E190^5.40^)^16–18^ is substituted by aspartate in both mGPR109A and rGPR109A, resulting in minor changes in the interaction network around this specific position (D187^5.40^ of mGPR109A). Despite these minimal differences, the binding modes of 6-fluoronicotinic acid and niacin are highly similar for human, mouse, and rat GPR109A receptors.

**Figure 7:**
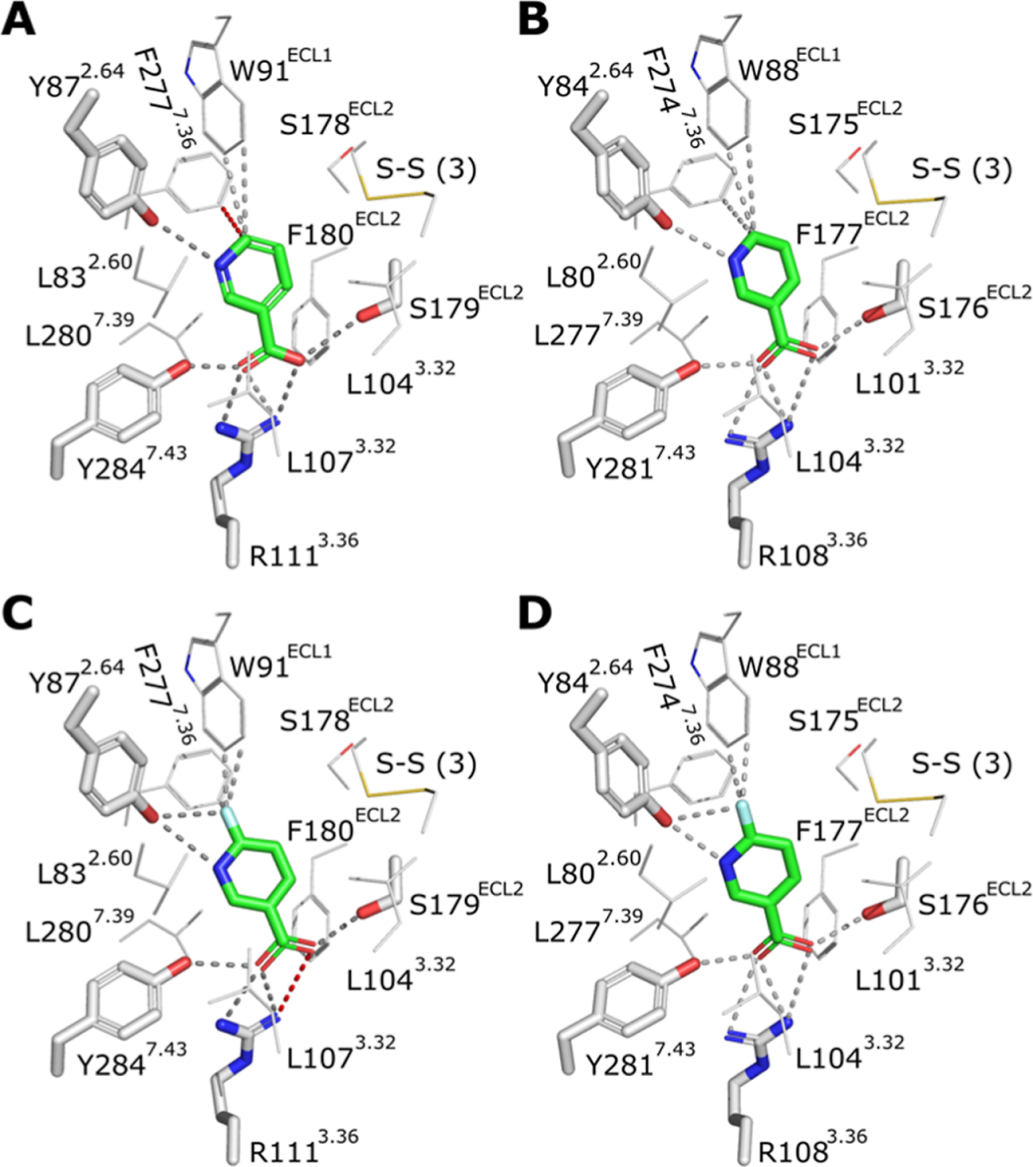
Molecular modeling of the binding of niacin and 6-fluoronicotinic acid to hGPR109A and mGPR109A. (A) Binding mode of niacin as in the cryoEM structure of hGPR109A (PDB ID 8J6P).^18^ (B) Binding mode of niacin based on docking to the mGPR109A homology model. (C) Binding mode of 6-fluoronicotinic acid based on docking to the cryoEM structure of hGPR109A. (D) Binding mode of 6-fluoronicotinic acid based on docking to the mGPR109A homology model. (A–D) The amino acid residues making hydrogen bonds (or ionic interactions) to niacin/6-fluoronicotinic acid (green) are shown as thick sticks; putative interactions are shown as dashed lines (distances < 4 Å, gray; 4.1 Å, red). The putative interactions of the pyridine ring of niacin or the fluorine atom of 6-fluoronicotinic acid with W91^ECL1^(hGPR109A)/W88^ECL1^(mGPR109A) and Y87^2.64^(hGPR109A)/Y84^2.64^(mGPR109A) are also indicated by dashed lines (distances < 4 Å, gray; 4.1 Å, red).

## 4 Discussion

MCT1 is highly expressed in several cancers and is a promising drug target for clinical treatment.^6^ MCT1 is also expressed in the BBB, providing opportunities for the development of brain tumor theranostics. In this work, we prepared [^18^F]­FNA using a straightforward method. The intracranial murine model of human glioblastoma was prepared using the patient-derived BT12 cells, which are grown as spheroids without the serum to preserve the glioblastoma stem cell like properties. [^18^F]­FNA was i.v. administered to mice, and the PET imaging performance was excellent and reproducible. [^18^F]­FNA accumulates rapidly in glioblastoma within a few minutes, at which TBRs become sufficiently high to clearly delineate tumors, and the fast kinetics do not require a long waiting time after tracer administration, thus being convenient in clinical practice. [^18^F]­FNA is stable both in vitro and in vivo, making it valuable for clinical applications.

In the presence of the MCT1 inhibitor AZD3965, even at a low dose (1.1 mg/kg), the [^18^F]­FNA uptake was blocked in both glioblastoma and healthy brain (Figure 4A). The blocking effect was also observed in several other organs (Table S1), including the heart, which is an MCT1-rich organ. These results indicate that [^18^F]­FNA can indeed be transported by MCT1. According to the TACs (Figure 4E), in the presence of a large excess amount of nonradiolabeled niacin (60 mg/kg), the [^18^F]­FNA uptake was significantly increased in glioblastoma and the healthy brain. This result aligns well with the theory that MCT1-mediated transport depends on the concentration gradient of substrates.^19^ When there was a large amount of niacin in the blood circulation, the transport into tissues and cells became more efficient. Interestingly, in the presence of a large excess of niacin, [^18^F]­FNA was better retained in the glioblastoma, as indicated by the TACs (Figure 4E). The finding of the niacin-enhanced [^18^F]­FNA tumor uptake and retention is of clinical relevance. Niacin is an essential nutrient that exists in the human body and is used as a medication for certain diseases. Our results suggest that the [^18^F]­FNA PET imaging performance is not compromised with the niacin intake, and patient fasting is not necessary before conducting [^18^F]­FNA PET. Moreover, the niacin-enhanced tumor uptake and retention are of high significance in the future development of niacin-based radiotherapeutic agents. Previously, in a [^11^C]­niacin PET imaging study in healthy mice,^10^ niacin preadministration reduced the [^11^C]­niacin uptake in several tissues. In our [^18^F]­FNA PET imaging with the glioblastoma-bearing mice, niacin preadministration increased the uptake of [^18^F]­FNA in the brain and other tissues (Table S1). [^18^F]­FNA is a fluorinated niacin analogue, and we do not anticipate that [^18^F]­FNA has identical biological properties as niacin or its isotopically labeled molecule [^11^C]­niacin. This is a similar case of glucose versus its fluorinated analogue 2-deoxy-2-[^18^F]­fluoroglucose ([^18^F]­FDG).

Niacin is a known agonist of GPR109A. To further explain why niacin preadministration can enhance the [^18^F]­FNA tumor uptake and retention, we cannot exclude the role of GRP109A activation by niacin. This prompted us to investigate the effect of GPR109A inhibition on the [^18^F]­FNA uptake. [^18^F]­FNA was cleared from the tumor and brain tissue significantly more slowly in the presence of mepenzolate bromide as a GPR109A inhibitor (Figure 4E). TACs with GPR109A inhibition had trends similar to those of GPR109A activation by niacin. GPR109A inhibition did not decrease the [^18^F]­FNA uptake, but changed the retention kinetics in both glioblastoma and healthy brain. These results show that both GPR109A activation and inhibition affect the [^18^F]­FNA tumor uptake and retention. We then performed an extensive in silico modeling study of [^18^F]­FNA–GPR109A interactions and found that [^18^F]­FNA indeed binds to GPR109A similarly to niacin itself across species in mice, rats, and humans. This also indicates that this preclinical study in mice has the potential to be translated to human use.

Glioblastoma has high histological and molecular heterogeneity, and several subtypes of tumor cells and cell clusters may coexist within the same tumor tissue.^20^ As a study limitation, we performed a PET evaluation of [^18^F]­FNA in glioblastomas prepared from only one subtype of tumor cells. We randomly selected five types of patient-derived glioblastoma cells, including BT12 cells, for molecular analysis by the Western blot, and MCT1 and GPR109A were detected in all cell types (Figure 6). Additionally, the MCT1 mRNA expression was identified in major subtypes of glioblastoma stem cells and tumor tissue structures. This finding holds promise for MCT1-mediated transport in PET imaging, warranting further studies.

## 5 Conclusion

[^18^F]­FNA was prepared using a straightforward radiosynthesis method with high radiochemical purity and exhibited excellent stability in vitro and in vivo. Patient-derived intracranial BT12 glioblastoma xenografts were clearly visualized by [^18^F]­FNA PET. [^18^F]­FNA quickly accumulated in glioblastoma and was predominantly excreted in the urinary tract. Mechanistically, our results revealed that MCT1 is a critical transporter mediating the [^18^F]­FNA into brain tumors. MCT1 blockade and preadministration of niacin or mepenzolate affect the [^18^F]­FNA tumor uptake or tumor retention. The underlying mechanisms warrant further investigation to facilitate the clinical translation of [^18^F]­FNA and the development of niacin-derived radiotherapeutic agents.

## Supplementary Material



## Notes

Animal study design, additional ex vivo biodistribution data, HPLC chromatographs of in vivo stability tests, and in silico homology modeling and docking analyses (PDF)

## Abbreviations

FNA: 6-fluoronicotinic acid
HPLC: high-performance liquid chromatography
MCT1: monocarboxylate transporter 1
PET: positron emission tomography
SUV: standardized uptake value
TAC: time–activity curve
% ID/g: percentage of injected dose per gram (of tissue)
