# Supplementary material for: Utilizing Monocarboxylate Transporter 1‑Mediated Blood–Brain Barrier Penetration for Glioblastoma Positron Emission Tomography Imaging with 6‑[18F]Fluoronicotinic Acid
Source: Mol Pharm. 2025 Jul 3;22(8):4819–30. doi: 10.1021/acs.molpharmaceut.5c00457 (PMC12326355; doi:10.1021/acs.molpharmaceut.5c00457)
Supplement: Supplementary file 1 [file mp5c00457_si_001.pdf]

Supporting Information

Utilizing monocarboxylate transporter 1-mediated blood–brain barrier penetration for glioblastoma PET imaging with 6-[^{18}F]fluoronicotinic acid

Pyry Dillemuth[1,2], Abiodun Ayo[3,4], Tomi T. Airenne[5], Petter Lövdahl[1,6], Emel Bakay[1,6], Xiaoqing Zhuang[1,2], Heidi Liljenbäck[1], Sami Tuomas Paunonen[1,2], Jonne Kunnas[1,6], Pauliina Filppu[3,4], Johan Rajander[1,7], Mark S. Johnson[5], Anne Roivainen[1,8,9,10], Tiina A. Salminen[5], Jessica M. Rosenholm[6], Pirjo Laakkonen[3,4,11], and Xiang-Guo Li*[1,2,8,9]

[1]Turku PET Centre, University of Turku, Turku FI-20520, Finland; [2]Department of Chemistry, University of Turku, Turku FI-20500, Finland; [3]Translational Cancer Medicine Research Program, Faculty of Medicine, University of Helsinki, Helsinki FI-00290, Finland; [4]iCAN Flagship Program, University of Helsinki, Helsinki FI-00290, Finland; [5]Structural Bioinformatics Laboratory and InFLAMES Research Flagship Center, Biochemistry, Faculty of Science and Engineering, Åbo Akademi University, Turku FI-20520, Finland; [6]Pharmaceutical Sciences Laboratory, Faculty of Science and Engineering, Åbo Akademi University, Turku FI-20520, Finland; [7]Accelerator Laboratory, Åbo Akademi University, Turku FI-20520, Finland; [8]Turku PET Centre, Turku University Hospital, Turku FI-20520, Finland; [9]InFLAMES Research Flagship, University of Turku, Turku FI-20520, Finland; [10]Turku Center for Disease Modeling, University of Turku, Turku FI-20520, Finland; [11]Laboratory Animal Centre, HiLIFE University of Helsinki, Helsinki FI-00290, Finland.

***Corresponding Author:** Associate Professor Xiang-Guo Li, PhD, Turku PET Centre, University of Turku, Kiinamyllynkatu 4-8, FI-20520 Turku, Finland; Phone: +358 50 4485069; E-mail: xiali@utu.fi

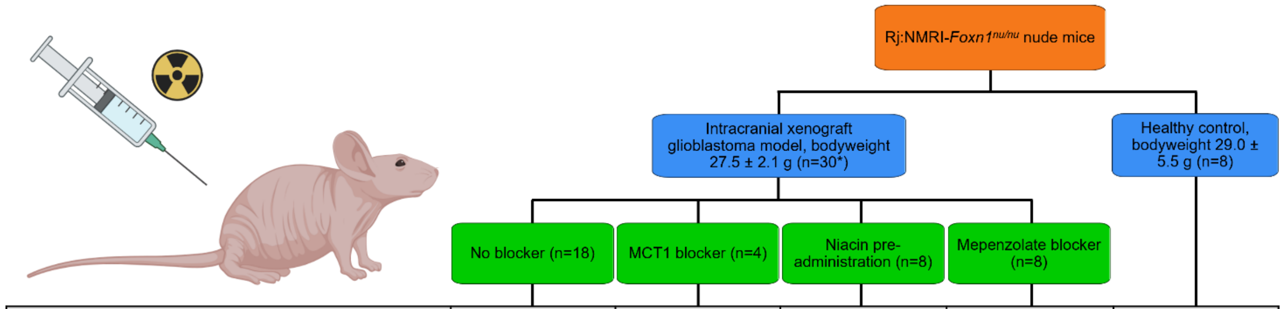

**Dynamic 60-minute PET-CT, [^{18}F]niacin**	**n=18**	**n=4**	**n=8**	**n=8**	**n=4**
Brain cryosectioning, autoradiography	n=8	n=4			
Ex vivo biodistribution	n=4	n=4	n=8	n=8	n=4
Metabolite analysis	n=4				

*Some animals were imaged twice during separate days.

SUPPLEMENTAL FIGURE 1. Animal study design.

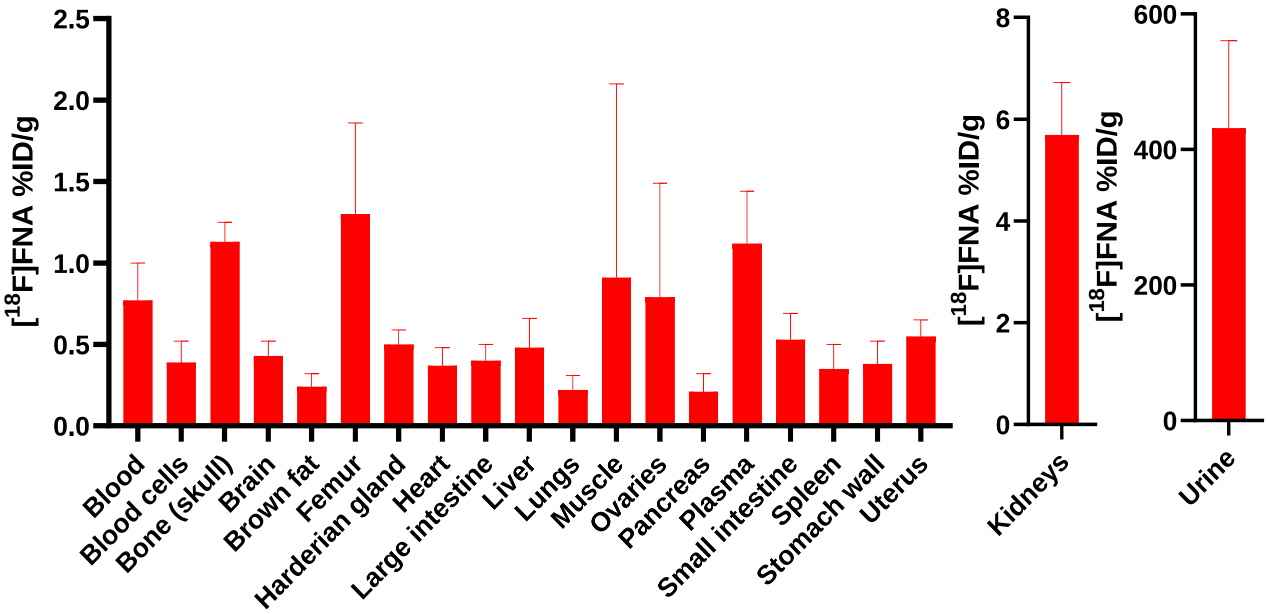

SUPPLEMENTAL FIGURE 2. *Ex vivo* biodistribution of [^{18}F]FNA at 60 minutes post-injection in mice with intracranial glioblastoma xenograft.

SUPPLEMENTAL TABLE 1

Ex vivo biodistribution of [^{18}F]FNA (Data expressed as %ID/g)

	Glioblastoma	Healthy control	MCT1 blocker	Mepenzolate 5 mg/kg	Mepenzolate 15 mg/kg	Niacin 15 mg/kg	Niacin 60 mg/kg
Blood *, **	0.77 ± 0.23	2.78 ± 0.47*** ↑	0.4 ± 0.25	0.95 ± 0.30	2.05 ± 0.43** ↑	1.53 ± 0.57* ↑	3.25 ± 0.35*** ↑
Blood cells **	0.39 ± 0.13	1.60 ± 0.33*** ↑	0.22 ± 0.15	0.50 ± 0,17	1.63 ± 0.92	0.84 ± 0.31* ↑	1.70 ± 0.29*** ↑
Bone (skull) **	1.13 ± 0.12	1.34 ± 0.12	1.26 ± 0.16	1.58 ± 0.32* ↑	2.62 ± 0.85* ↑	3.6 ± 0.62** ↑	2.05 ± 0.41** ↑
Brain *, *	0.43 ± 0.09	1.05 ± 0.13*** ↑	0.1 ± 0.02** ↓	0.57 ± 0.19	1.27 ± 0.45* ↑	0.88 ± 0.30* ↑	1.64 ± 0.21*** ↑
Brown fat *	0.24 ± 0.08	0.79 ± 0.21** ↑	0.1 ± 0.05* ↓	0.28 ± 0.07	0.87 ± 0.55	0.48 ± 0.13* ↑	0.94 ± 0.31** ↑
Femur (bone + marrow)	1.30 ± 0.56	1.36 ± 0.16	1.23 ± 0.21	1.40 ± 0.28	2.08 ± 0.59	2.37 ± 0.55* ↑	1.77 ± 0.15
Harderian glands *	0.50 ± 0.09	1.71 ± 0.48* ↑	0.32 ± 0.08* ↓	0.79 ± 0.35	1.86 ± 1.22	1.10 ± 0.26** ↑	1.93 ± 0.57* ↑
Heart *	0.37 ± 0.11	1.37 ± 0.27*** ↑	0.18 ± 0.06* ↓	0.45 ± 0.16	1.11 ± 0.72	0.63 ± 0.14* ↑	1.19 ± 0.30** ↑
Kidneys	5.69 ± 1.03	13.34 ± 4.18* ↑	2.48 ± 1.55* ↓	8.40 ± 2.95	28.58 ± 25.16	11.10 ± 2.62** ↑	9.33 ± 1.92* ↑
Large intestine *	0.40 ± 0.10	1.58 ± 0.29*** ↑	0.36 ± 0.32	0.62 ± 0.27	1.38 ± 0.77	0.91 ± 0.30* ↑	1.58 ± 0.39** ↑
Liver *	0.48 ± 0.18	2.07 ± 0.65** ↑	0.25 ± 0.08	0.65 ± 0.21	1.42 ± 0.56* ↑	1.09 ± 0.46	1.61 ± 0.24*** ↑
Lungs	0.22 ± 0.09	0.87 ± 0.40* ↑	0.10 ± 0.03* ↓	0.27 ± 0.20	0.42 ± 0.25	0.40 ± 0.20	0.59 ± 0.23* ↑
Muscle **	0.91 ± 1.19	1.48 ± 0.86	0.15 ± 0.06	0.40 ± 0,13	1.10 ± 0.56	0.59 ± 0.19	1.27 ± 0.28
Ovaries *	0.79 ± 0.70	1.61 ± 0.46	0.29 ± 0.20	0.63 ± 0.30	1.62 ± 1.23	1.00 ± 0.35	1.71 ± 0.39
Pancreas *	0.21 ± 0.11	1.22 ± 0.33** ↑	0.18 ± 0.08	0.41 ± 0.18	1.05 ± 0.80	0.61 ± 0.19** ↑	1.16 ± 0.32** ↑
Plasma **	1.12 ± 0.32	3.93 ± 0.65*** ↑	0.57 ± 0.34	1.35 ± 0.42	3.96 ± 2.12	2.17 ± 0.84	4.77 ± 0.40*** ↑
Small intestine	0.53 ± 0.16	2.34 ± 0.33*** ↑	0.23 ± 0.10* ↓	0.69 ± 0.26	1.82 ± 0.98	1.04 ± 0.35* ↑	1.71 ± 0.45** ↑
Spleen	0.35 ± 0.15	1.60 ± 0.59* ↑	0.20 ± 0.07	0.49 ± 0.15	1.21 ± 0.80	0.82 ± 0.28* ↑	1.25 ± 0.32** ↑
Stomach wall *	0.38 ± 0.14	1.58 ± 0.42** ↑	0.18 ± 0.09* ↓	0.55 ± 0.20	1.44 ± 0.93	0.83 ± 0.34* ↑	1.49 ± 0.25*** ↑
Urine *	431.43 ± 129.28	166.46 ± 287.35	461.38 ± 115.85	561.44 ± 278.98	238.66 ± 181.28	627.06 ± 275.57	182.37 ± 71.31* ↑
Uterus	0.55 ± 0.10	2.09 ± 0.36** ↑	0.59 ± 0.39	0.78 ± 0.36	1.60 ± 0.83	1.41 ± 0.67	2.00 ± 0.38** ↑

The number of mice in each group was four (n=4).
All the mice were with glioblastoma except the healthy controls.
P-values compared to the glioblastoma group: *P<0.05, **P<0.01, ***P<0.001.
P-values between the mouse groups administrated with mepenzolate at different doses: *P<0.05
P-values between the mouse groups administrated with niacin at different doses: *P<0.05, **P<0.01

SUPPLEMENTAL TABLE 2

Ex vivo biodistribution of [^{18}F]FNA (Data expressed as %ID)

	Glioblastoma	Healthy Control	MCT1 blocker	Mepenzolate 5 mg/kg	Mepenzolate 15 mg/kg	Niacin 15 mg/kg	Niacin 60 mg/kg
Brain *,*	0.19 ± 0.05	0.48 ± 0.07*** ↑	0.06 ± 0.02** ↓	0.24 ± 0.08	0.62 ± 0.25* ↑	0.39 ± 0.13* ↑	0.78 ± 0.12*** ↑
Harderian Glands *	0.02 ± 0.00003	0.06 ± 0.03* ↑	0.01 ± 0.00002** ↓	0.02 ± 0.01	0.06 ± 0.03	0.03 ± 0.01* ↑	0.05 ± 0.01*** ↑
Heart **	0.05 ± 0.02	0.24 ± 0.05*** ↑	0.03 ± 0.01	0.06 ± 0.03	0.13 ± 0.08	0.08 ± 0.01* ↑	0.15 ± 0.03** ↑
Kidneys	2.29 ± 0.52	6.91 ± 2.64* ↑	1.08 ± 0.79* ↓	3.11 ± 1.13	11.91 ± 9.27	4.00 ± 0.91* ↑	4.07 ± 0.47** ↑
Liver *,***	0.38 ± 0.12	3.10 ± 1.18* ↑	0.35 ± 0.18	0.59 ± 0.48	1.87 ± 0.74* ↑	0.71 ± 0.27	2.24 ± 0.26*** ↑
Ovaries *,*	0.01 ± 0.00005	0.04 ± 0.02* ↑	0.01 ± 0.00003	0.01 ± 0.00003	0.03 ± 0.01* ↑	0.02 ± 0.01	0.03 ± 0.01** ↑
Uterus	0.05 ± 0.01	0.35 ± 0.12* ↑	0.06 ± 0.03	0.10 ± 0.10	0.14 ± 0.12	0.18 ± 0.13	0.17 ± 0.09

The number of mice in each group was four (n=4).
Apart from the healthy control group, all mice were bearing intracranial xenografts of glioblastoma. P-values compared to the glioblastoma group: *P<0.05, **P<0.01, ***P<0.001.
P-values between the mouse groups administrated with mepenzolate at different doses: *P<0.05
P-values between the mouse groups administrated with niacin at different doses: *P<0.05, **P<0.01

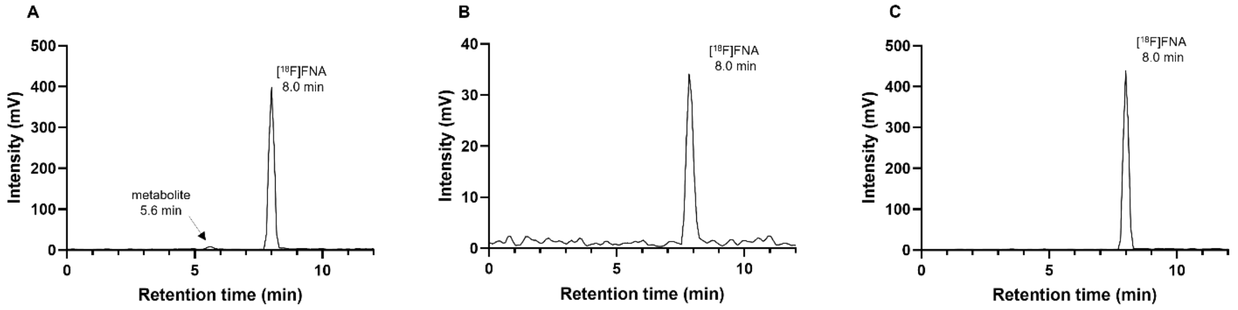

SUPPLEMENTAL FIGURE 3. HPLC analysis of in vivo stability of [^{18}F]FNA at 60 minutes post-administration. (A) Radio-HPLC chromatogram of the protein-free plasma sample. Intact [^{18}F]FNA was observed at a retention time of 8.0 minutes with trace amounts of metabolite observed at a retention time of 5.6 min. (B) Radio-HPLC chromatogram of the brain homogenate sample. Intact [^{18}F]FNA peak was observed at a retention time of 8.0 minutes. (C) Radio-HPLC chromatogram of the [^{18}F]FNA reference standard.

Homology modeling and docking analyses

The interactions between human, rat and mouse GPR109A (also known as hydroxycarboxylic acid receptor 2 or niacin receptor 1), and 6-fluoronicotinic acid (non-radioactive form of [^{18}F]FNA) were studied using homology modeling and docking analyses. Coordinates for human GPR109A were from the Protein Data Bank (PDB code 8J6P).[1] The structure files for two conformers (Conformer3D_COMPOUND_CID_242819.sdf and Conformer3D_COMPOUND_CID_242819-2.sdf) of 6-fluoronicotinic acid were from PubChem (https://pubchem.ncbi.nlm.nih.gov/compound/6-

Fluoronicotinic-acid)[2] and for niacin (NIO_ideal.sdf) from RCSB PDB (https://www.rcsb.org/ligand/NIO).[3] For docking, the WEBINA server (1.0.5; https://durrantlab.pitt.edu/webina/)[4] was used; in each docking, the box size (140, 126, 91) and center of the box (25, 25, 25) were the same, and the server was used to prepare the human (hGPR109A; PDB ID 8J6P), rat (rGPR109A; homology model described here) and mouse (mGPR109A; homology model described here) receptors, as well as the ligands (niacin and 6-fluoronicotinic acid), for the docking experiments.

Homology models of mouse and rat GPR109A were created using Modeller 10.5.[5] The human cryoEM GPR109A structure (PDB code 8J6P; residues 8-302 of chain E) was chosen as the structural template since, to our knowledge, it is the highest resolution (2.55 Å) experimental structure of GPR109A in an active conformation and in a complex with niacin. As G protein-coupled receptors (GPCRs) are known to adapt to bind a wide range of different small molecule ligands, we considered it to be important to use a structural template for modeling, which contains a ligand as similar as possible to the ligands to be analyzed by docking. Multiple sequence alignment (MSA) of human (UniProt ID Q8TDS4),[6] rat (Q80Z39) and mouse (Q9EP66) GPR109A sequences for homology modeling was created using ClustalW (default settings)[7] incorporated in MEGA 11[8] (Supplemental Figure 4) and visualized using ESPript 3.0.[9] The created models of rGPR109A and mGPR109A included all residues of the full-length sequences but for the structural analysis only the residues matching positions 8-302 of the hGPR109A structural template were considered. All figures representing structural information were created using PyMOL (The PyMOL Molecular Graphics System, Version 3.0 Schrödinger, LLC.) and Inkscape (https://inkscape.org).

In silico analyses of 6-fluoronicotinic acid binding to human GPR109A and its rat and mouse homologs

We created the homology models of the rat (rGPR109A) and mouse receptor (mGPR109A) to understand the putative differences in the binding of both niacin and 6-fluoronicotinic acid among the species, and the putative effect on PET imaging. The models are overall very similar to each other as well as in comparison to the cryoEM structure of the human receptor (hGPR109A) in complex with niacin (PDB code 8J6P,[1] Supplemental Figure 5). All niacin-interacting residues are conserved and most of the amino acid differences are located on the “top” of the receptor in regions facing the extracellular space that close the orthosteric binding pocket of GPR109A – none of them directly affect niacin binding; the putative effect of these differences is out of the scope of this study. However, E190$^{5.40}$ (the superscript refers to Ballesteros-Weinstein numbering)[10] of hGPR109A, a residue involved in major structural reorganization during receptor activation,[11,12] is substituted by aspartate both in mGPR109A and rGPR109A (Supplemental Figures 6-8) and will be discussed in detail below. Since the rGPR109A and mGPR109A models are almost identical, we focused our analyses mainly on the mouse and human structures.

The ligand-binding pocket of hGPR109A is closed unlike for canonical class A G protein-coupled receptors and the residues involved in niacin binding are conserved between hGPR109A and

mGPR109A (Supplemental Figure 4). However, in close proximity to the ligand-binding pocket (Supplemental Figure 6), glutamic acid E190$^{5.40}$ of hGPR109A is replaced with an aspartate residue (D187$^{5.40}$) in mGPR109A/rGPR109A (Supplemental Figure 7): E190$^{5.40}$ forms a salt bridge with arginine R251$^{6.55}$ in the active form of hGPR109A, whereas in the inactive form a hydrogen bond is formed between R251$^{6.55}$ and serine S181^{ECL2} (Supplemental Figure 8).[11] Comparison of the interactions of E190$^{5.40}$ (hGPR109A[1]) and D187$^{5.40}$ (mGPR109A; homology model described here) suggests that similarly to hGPR109A the shorter D187$^{5.40}$ side chain of mGPR109A may stabilize the active form of the receptor and form a salt bridge with R248$^{6.55}$. Furthermore, due to the different conformer of R248$^{6.55}$ (mGPR109A), R248$^{6.55}$ can form hydrogen bond to the main-chain oxygen atom of S178^{ECL2} unlike in hGPR109A. The configuration of putative weak interactions is also different between hGPR109A and mGPR109A.

Comparison of the binding of niacin and 6-fluoronicotinic acid to hGPR109A and mGPR109A

We docked 6-fluoronicotinic acid into the cryoEM structure of hGPR109A[1] and to the homology model of mGPR109A, and niacin into the mGPR109A structure (Figure 7). As a control, niacin was also docked back to the hGPR109A-niacin complex structure (PDB ID 8J6P) after deleting the ligand from the cryoEM structure – one of the top-ranked niacin poses bound nearly identically as in the published cryoEM structure[1] (data not shown). Based on the docking analyses, the binding modes of niacin and 6-fluoronicotinic acid to hGPR109A and mGPR109A are very similar, as might be expected given that all residues involved in ligand binding are identical. The carboxyl group of both ligands is critical for interaction with R111$^{3.36}$(hGPR109A)/R108$^{3.36}$(mGPR109A), which has been reported in earlier studies.[1,11,12] Based on visual inspection of the docking results (all conformers), the binding mode of niacin and 6-fluoronicotinic acid in which the pyridinic nitrogen of the ligand is hydrogen bonded to Y87$^{2.64}$(hGPR109A)/Y84$^{2.64}$(mGPR109A) is likely to be preferred over an alternative observed binding mode in which the pyridinic nitrogen is facing S178^{ECL2}(hGPR109A)/S175^{ECL}(mGPR109A), a binding mode also reported by e.g. Zhao and coworkers.[12] The interactions of niacin and 6-fluoronicotinic acid with the hGPR109A and mGPR109A receptors are shown in detail in Figure 7. The 6-fluoronicotinic acid ligand has an extra fluorine atom attached to the pyridine ring in comparison to niacin. The fluorine atom is within bonding distance of W91^{ECL1} in hGPR109A (Fig. 6c) and W88^{ECL1}in mGPR109A (Fig. 6d); the proximity to a tryptophan residue may have an influence in PET imaging.

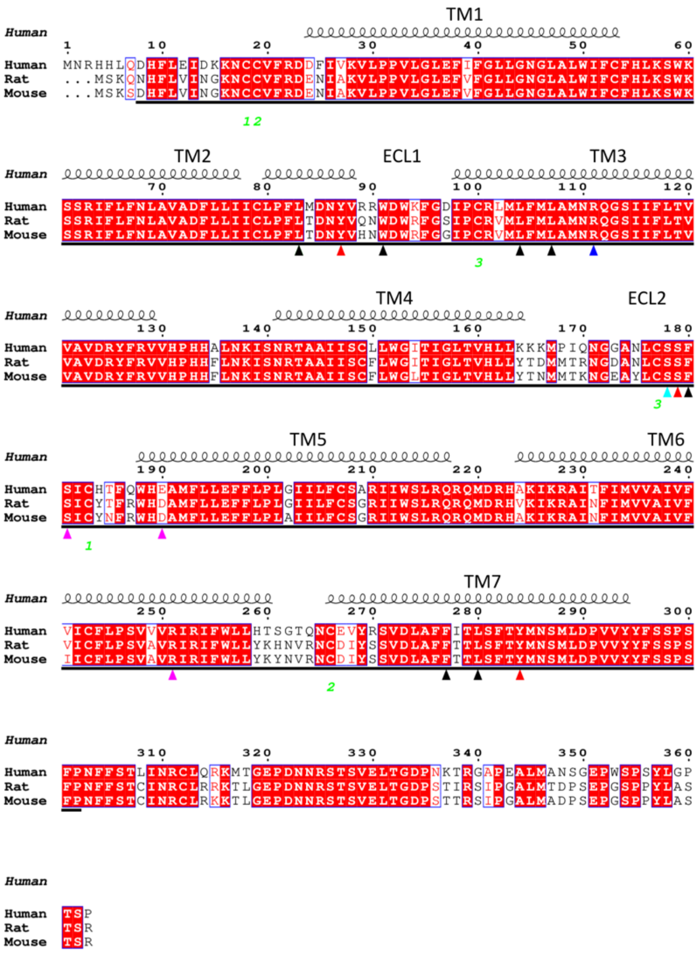

SUPPLEMENTAL FIGURE 4. Sequence alignment of human (UniProt ID Q8TDS4), rat (Q80Z39) and mouse (Q9EP66) GPR109A sequences. The secondary structure elements matching transmembrane helices 1-7 (TM1-TM7) and extracellur loop 1-2 (ECL1-2) are shown according to the cryoEM structure of human GPR109A (PDB ID 8J6P; chain E covering residues 8-302; black line under the sequences; residue 302 in 8JP6 is asparagine); this structure was used as the template in homology modeling. The alignment was created using ClustalW[7] in MEGA 11[8] and visualized using ESPript 3.0.[9] R111$^{3.36}$ crucial for ligand binding is depicted by a blue triangle; the hydrophobic residues L83$^{2.60}$, W91^{ECL1}, L104$^{3.29}$, L107$^{3.32}$, F180^{ECL2}, F277$^{7.36}$ and L280$^{7.39}$ at the ligand-binding pocket by a black triangle; Y87$^{2.64}$, S179^{ECL2} and Y284$^{7.43}$ hydrogen bonded to niacin (nicotinic acid; PDB code 8JP6)[1] or 6-fluoronicotinic acid (homology models described here) by a red triangle; S178^{ECL2} reported in some structures to be hydrogen bonded to niacin[12] by a cyan triangle; and S181^{ECL2}, E190$^{5.40}$ and R251$^{6.55}$ near ligand-binding pocket and suggested to face major structural reorganization during receptor activation[11] by magenta triangles. All the depicted residues except E190$^{5.40}$ are conserved between human and mouse/rat. The disulfide bridges are indicated with green numbers.

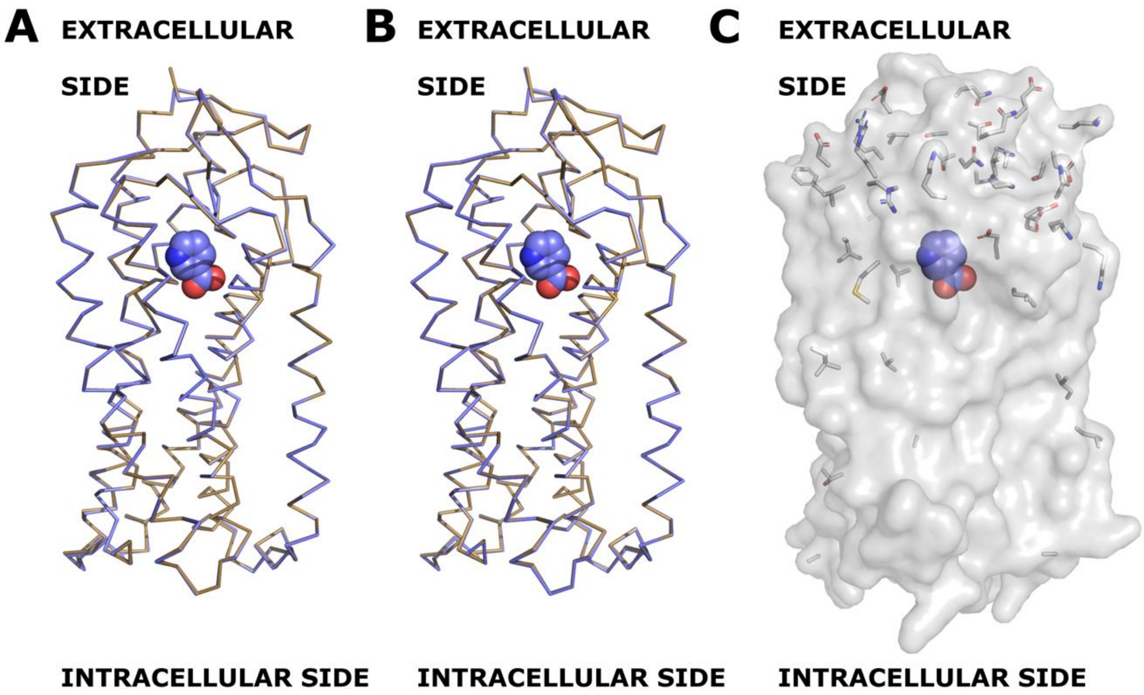

SUPPLEMENTAL FIGURE 5. Comparison of the cryoEM structure (blue ribbon) of hGPR109A in complex of niacin (PDB ID 8J6P) to the homology model (gold ribbon) of (A) rGPR109A and (B) mGPR109A. The niacin ligand of the hGPR109A structure is shown as spheres. (C) Surface presentation of hGPR109A showing as sticks the location of the nonconserved residues (human vs. rat/mouse). GPR109A is also known as the hydroxycarboxylic acid receptor 2 or niacin receptor 1.

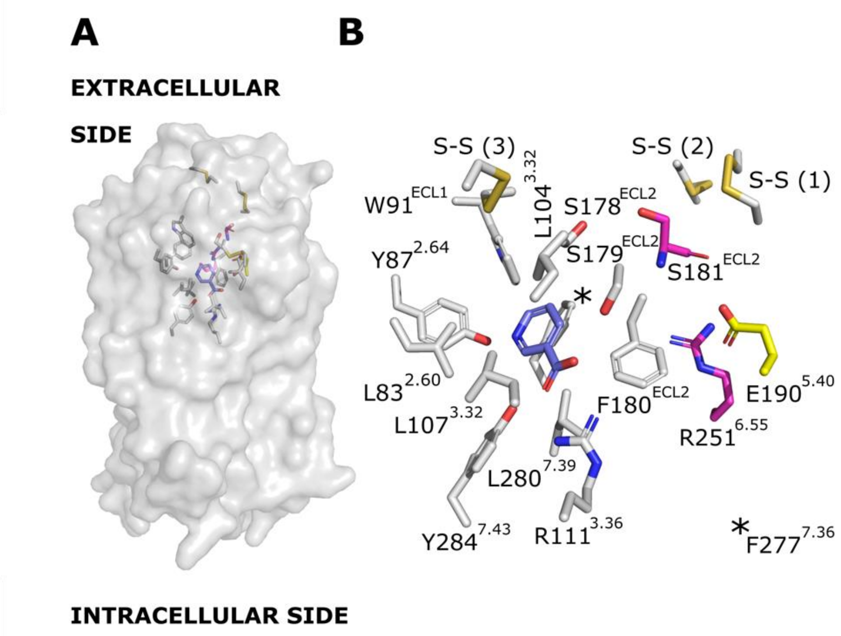

SUPPLEMENTAL FIGURE 6. Ligand-binding site of GPR109A. (A) Surface presentation of the human cryoEM structure with a bound niacin (blue) molecule (PDB ID 8J6P) pinpointing the location of the

ligand-binding site. (B) Residues in contact with niacin (grey carbon atoms) and residues important in remodeling the ligand-binding site during receptor activation (E190$^{5.40}$ yellow and R251$^{6.55}$/S181^{ECL2} magenta carbon atoms) are shown as sticks. In mGPR109A, D187$^{5.40}$ is equivalent to E190$^{5.40}$ of hGPR109A (Supplemental Figure 7). The disulfide bridges (S-S) important for the compact conformation of the extracellular side are numbered as in Supplemental Figure 4.

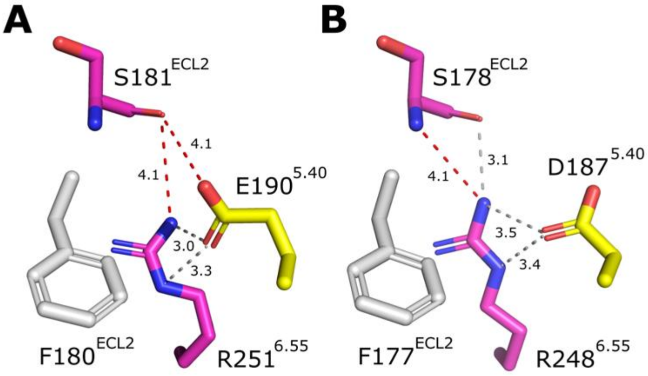

SUPPLEMENTAL FIGURE 7. Residues involved in remodeling the ligand-binding site during receptor activation. Interactions of (A) E190$^{5.40}$ of hGPR109A (PDB ID 8J6P) and (B) D187$^{5.40}$ of mGPR109A (homology model described here). Coloring scheme as in Supplemental Figure 6. Strong interactions (hydrogen bonds and ionic interactions; distance < 4 Å) are indicated with gray dashed lines and putative weak interactions (4.1 Å) with red dashed lines. Distances are shown in Ångströms (Å). F180^{ECL2} – in contact with niacin in hGPR109A – is shown to pinpoint the proximity of the key residues to the ligand-binding site.

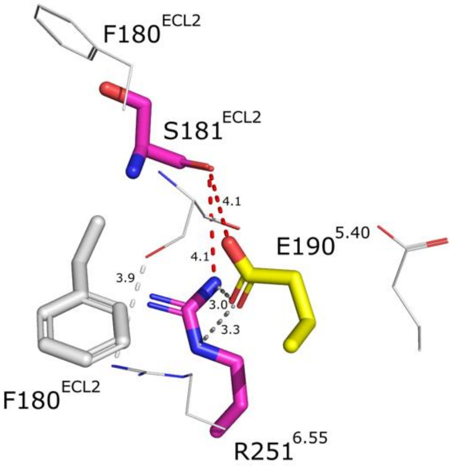

SUPPLEMENTAL FIGURE 8. Comparison of three key residues for receptor activation in the active (thick sticks; PDB ID 8J6P)[1] and inactive (thin sticks; PDB ID 7ZL9)[11] form of the hGPR109A receptor.

The residue equivalent to E190$^{5.40}$ (yellow) of hGPR109A is D187$^{5.40}$ in mGPR109A (Supplemental Figure 4 and 7) and alterations in interactions with residues R251$^{6.55}$ and S181^{ECL2} (magenta) are critical for activation. Strong interactions (hydrogen bonds and ionic interactions; distance < 4 Å) are indicated with gray dashed lines and putative weak interactions (4.1 Å) with red dashed lines. Distances are shown in Ångströms (Å). F180^{ECL2} in contact with niacin in hGPR109A (PDB ID 8J6P) is shown to pinpoint the proximity of the key residues to the ligand-binding site.

REFERENCES

1. Mao C, Gao M, Zang SK, et al. Orthosteric and allosteric modulation of human HCAR2 signaling complex. *Nat Commun.* 2023;14:7620.
2. Kim S, Chen J, Cheng T, et al. PubChem 2023 update. *Nucleic Acids Res*. 2023;51:D1373-D1380.
3. Berman HM, Westbrook J, Feng Z, et al. The Protein Data Bank. *Nucleic Acids Res*. 2000;28:235-242.
4. Kochnev Y, Hellemann E, Cassidy KC, Durrant JD. Webina: an open-source library and web app that runs AutoDock Vina entirely in the web browser. *Bioinform.* 2020;36:4513-4515.
5. Šali A, Blundell TL. Comparative protein modelling by satisfaction of spatial restraints. *J Mol Biol.* 1993;234:779-815.
6. UniProt C. UniProt: the Universal Protein Knowledgebase in 2025. *Nucleic Acids Res*. 2025;53:D609-D617.
7. Thompson JD, Higgins DG, Gibson TJ. Clustal-W - Improving the Sensitivity of Progressive Multiple Sequence Alignment through Sequence Weighting, Position-Specific Gap Penalties and Weight Matrix Choice. *Nucleic Acids Res.* 1994;22:4673-4680.
8. Stecher G, Tamura K, Kumar S. Molecular Evolutionary Genetics Analysis (MEGA) for macOS. *Mol Biol Evol*. 2020;37:1237-1239.
9. Robert X and Gouet P (2014) Deciphering key features in protein structures with the new ENDscript server. *Nucleic Acids Res*. 2014;42(W1):W320-W324.
10. Ballesteros JA & Weinstein H. Integrated methods for the construction of three-dimensional models and computational probing of structure-function relations in G protein-coupled receptors. *Methods Neurosci*. 1995;25:366–428.
11. Yang Y, Kang HJ, Gao R, et al. Structural insights into the human niacin receptor HCA2-G(i) signalling complex. *Nat commun*. 2023;14:1692.
12. Zhao C, Wang H, Liu Y, et al. Biased allosteric activation of ketone body receptor HCAR2 suppresses inflammation. *Mol Cell* 2023;83:3171–3187.
